# Perceptions and Acceptability of *Artemisia annua* Herbal Tea for Malaria Treatment: A Qualitative Study in Kalima Health Zone, Maniema, Democratic Republic of the Congo

**DOI:** 10.3390/healthcare14172740

**Published:** 2026-08-27

**Authors:** Jerome Munyangi wa Nkola, Pierre Akilimali Zalagile, Hendrick Lukuke Mbutshu, Spartacus Kabala Munyemo, Imani Ramazani Bin Eradi, Alioune Camara

**Affiliations:** 1 Ecole Doctorale en Sciences de la Vie, Santé et Environnement, Programme Doctoral en Santé Publique, Faculté des Sciences et Techniques de la Santé, Département des Sciences Médicales, Université Gamal Abdel Nasser de Conakry, Conakry BP 1147, Guinea; 2 Faculté de Médecine, Université de Kolwezi, Kolwezi BP 453147, Democratic Republic of the Congo; 3 Department of Nutrition, Kinshasa School of Public Health, University of Kinshasa, Kinshasa BP 11850, Democratic Republic of the Congo; 4 Patrick Kayembe Research Center, Kinshasa School of Public Health, University of Kinshasa, Kinshasa BP 11850, Democratic Republic of the Congo; 5 Ecole de Santé Publique, Université de Lubumbashi, Lubumbashi BP 1825, Democratic Republic of the Congo; 6 Secrétariat Général Académique, Université de Kindu, Kindu BP 122, Democratic Republic of the Congo; 7 Domaine de Pedagogie et Didactique des Disciplines, Universite Pedagogique Nationale, Kinshasa BP 8815, Democratic Republic of the Congo; 8 Nursing Sciences Research Chair, Laboratory of Education and Health Practices, ‘UFR SMBH’, University Paris, F-93017 Bobigny, France; 9 Nursing Sciences Section, Higher Institute of Medical Techniques of Kindu, Kindu BP 304, Democratic Republic of the Congo; 10 Department of Public Health-Epidemiology, Faculty of Sciences and Health Techniques, Gamal Abdel Nasser University, Conakry BP 1147, Guinea; 11 National Malaria Control Programme, Conakry BP 6339, Guinea

**Keywords:** Malaria, *Artemisia annua*, health belief model, behavioral and social drivers, community therapeutic behavior under supply chain failure, Democratic Republic of the Congo

## Abstract

**Highlights:**

**What are the main findings?**

- The adoption of *Artemisia annua* infusion in the Kalima Health Zone is a pragmatic “Structural Survival” strategy triggered by a dual failure in the formal health system: the physical absence of drugs (stockouts) and the erosion of trust due to a “Quality Vacuum” of substandard synthetic antimalarials.
- While end-users demonstrate high cognitive confidence in the plant’s efficacy and “purity,” biomedical providers express severe “posological anxieties” regarding the lack of standardization and the biological risk of sub-therapeutic dosing in uncalibrated home preparations.

**What are the implications of the main findings?**

- To safeguard the long-term efficacy of Artemisinin-based Combination Therapies, public health authorities must prioritize closing the “Quality Vacuum” of substandard medicines to prevent the selection and westward spread of Kelch 13 resistance markers already documented in neighboring East African regions.
- Integrating *Artemisia* into national policy requires transitioning from artisanal infusions to standardized pharmaceutical forms (tablets) while strategically engaging with both traditional community leaders and modern digital religious networks to combat health misinformation.

**Abstract:**

**Background:** Malaria is a significant public health challenge in the Democratic Republic of the Congo. In conflict-affected areas, access to quality-assured ACTs is restricted by chronic stockouts and circulation of substandard and falsified antimalarials. This study investigates perceptions and social acceptability of *Artemisia annua* herbal tea in the Kalima Health Zone. **Methods:** Distinct from the prior provider-arm cross-sectional survey of the same program in the Kalima Health Zone which sampled only biomedical healthcare workers (n = 337), this exploratory qualitative study adopted a socio-anthropological design across three stakeholder strata (community users, biomedical providers, opinion leaders). Maximum-variation purposive sampling recruited 30 informants across five health areas (11 end-users, 11 providers, 8 opinion leaders). Sample-size adequacy was assessed against Malterud’s information power framework; data collection continued until operational thematic saturation. Hybrid inductive–deductive thematic content analysis followed COREQ reporting. **Results:** End-users demonstrated high willingness, driven by accessibility, perceived three-day recovery, and avoidance of unreliable ACTs. Biomedical providers voiced intense posological anxieties over uncalibrated raw biomass and sub-therapeutic dosing. Behavioral patterns were shaped by socio-religious norms and digitally mediated religious discourse. A unanimous demand for industrial galenic transformation of raw biomass into calibrated tablets emerged across all groups. **Conclusions:** This study does not validate the clinical efficacy, safety, posological adequacy, or resistance-attenuation potential of *Artemisia annua* herbal tea. It documents a transversal household-level consensus, across the three strata and five health areas, that *Artemisia annua* herbal tea adoption responds to documented double ACT supply chain failure. A unanimous demand for industrial galenic transformation emerged across all stakeholder groups.

## 1. Introduction

Malaria persists as one of the most significant global public health challenges. The World Health Organization’s 2025 World Malaria Report situates the disease within a structurally alarming convergence of biological and financial pressures in sub-Saharan Africa. After a 25.6% decrease in case incidence between 2000 and 2015 (from 79.4 to 59.0 cases per 1000 population at risk), incidence has risen by approximately 8.5% between 2015 and 2024 [1]. The WHO African Region continues to bear approximately 94% of the global case burden and 95% of total global mortality [2]. Four biological threats now operate simultaneously across the continent: partial resistance to artemisinin in *Plasmodium falciparum*, parasites capable of evading WHO-recommended rapid diagnostic tests, widespread pyrethroid resistance in *Anopheles* vector populations, and the geographic expansion of *Anopheles stephensi* into previously unoccupied urban settings [1,2]. Compounding these biological threats, external financing for malaria programs has declined sharply, eliminating the fiscal headroom that previously buffered control programs against emergent resistance [2]. Within this high-burden landscape, the Democratic Republic of the Congo remains a major epicenter, contributing approximately 13% of global malaria cases and 11% of global malaria deaths according to recent surveillance evidence [2]. Resistance emergence and the simultaneous erosion of diagnostic, vector-control, and financing capacity are no longer independent threats but are operating as a single coupled system [2].

This chronic health crisis is compounded by macro-level structural and geopolitical vulnerabilities. Protracted instability has affected the eastern provinces of the DRC, which has pushed the formal healthcare infrastructure toward collapse [3]. These instabilities frequently disrupt transportation routes, causing chronic interruptions in the pharmaceutical supply chain [4]. Peripheral health zones in the Maniema Province often face critical shortages of essential biomedical countermeasures, a pattern described across South Kivu as the tyranny of empty shelves [5].

Yet the challenge is not limited to physical unavailability. A quality concern characterizes the regional pharmaceutical market: studies in Bukavu have documented that a significant proportion of antimalarials, including quinine and artemether-lumefantrine, fail to meet essential quality standards [6,7]. A separate Kinshasa-based study documented non-compliance patterns in pediatric medicines supplied through informal private wholesalers [8], and a national survey covering eight cities confirmed non-compliance in artemether-lumefantrine formulations [9]. Across the SADC region, substandard and falsified medical products have been formally identified as a continental priority [10], with validated HPLC and UV-Vis analyses conducted in the DRC confirming that the country faces a generalized problem affecting antimicrobial therapeutic classes [11]. When this documented regional evidence is set against chronic stockouts of essential child health medicines in the DRC [4], the convergence of physical and quality concerns becomes the empirical backdrop against which populations in conflict-affected provinces navigate therapeutic decisions.

A foundational distinction must be clearly stated: this study is explicitly not a clinical or pharmacological validation of *Artemisia annua* herbal tea. Public health institutions emphasize the significant pharmacological risks associated with raw biomass utilization, classifying it as an unstandardized monotherapy whose use is currently discouraged; artemisinin-based combination therapies remain the only validated first-line molecules for uncomplicated malaria [12,13]. Variable, sub-therapeutic doses of artemisinin through uncalibrated biomass create intense selection pressure on *Plasmodium falciparum*, which has been linked to the emergence of resistance markers such as Kelch 13 propeller mutations documented in neighboring East African countries [14,15,16]. Recent surveillance also documents *Artemisia*-independent inhibitory activity against different *Plasmodium* stages including relapse-causing hypnozoites [17], emphasizing that any standardized formulation would require rigorous pharmacological characterization, and that non-standardized infusions pose additional risks. The WHO-cited Ugandan medicinal plant evidence base further underscores that documented efficacy in standardized extracts cannot be presumed to transfer to artisanal teas [18].

The present study is anchored in the Kalima Health Zone of the Maniema Province, deliberately selected because it concentrates, within a single delimited territory, the structural variables hypothesized to drive therapeutic substitution toward *Artemisia annua*. Three convergent rationales justify this site of inquiry. First, Maniema Province lies within a high-burden context consistent with the DRC’s contribution of approximately 12% of global malaria cases and 11% of global malaria deaths nationally [17]. Second, the Kalima Health Zone is severely geographically enclaved: dense forest cover and the Ulindi River system fragment the district and isolate, in particular, the southeastern health areas, a configuration that recurs across supply chain analyses of inland eastern DRC [3,4]. Third, the Maniema Province is affected by protracted instability in the eastern DRC, whose impact on overland transportation has been documented to generate chronic disruptions in pharmaceutical distribution, a pattern characterized across South Kivu as the tyranny of empty shelves [3,5]. In Kalima, these three conditions operate simultaneously, producing the structural configuration within which the documented therapeutic plasticity of endemic populations can be empirically observed.

This paper operates strictly as a socio-behavioral investigation of perceptions, beliefs, and behaviors under documented systemic constraints. By adopting the integrated Health Belief Model and the WHO Behavioral and Social Drivers framework [18,19], the study decodes how individuals navigate health choices. Descriptive ethnobotanical studies remain urgently needed for the documentation of traditional knowledge in fragile contexts [20], and qualitative research is required to understand how communities operationalize botanical resources when formal health systems are incapacitated [21]. To address this empirical gap, this study investigates the perceptions and acceptability of *Artemisia annua* herbal tea within the enclaved health areas of the Kalima Health Zone.

*Distinction from prior work of the same research program*. A recent study by members of the same research program, conducted in the same Kalima Health Zone of Maniema Province, examined perceptions and acceptability of *Artemisia annua* herbal tea specifically among biomedical healthcare workers (provider survey; n = 337) [22]. The present qualitative study is formally independent of that prior cross-sectional survey. Three explicit differences warrant emphasis. First, the present study recruits three stakeholder strata community end-users (n = 11), biomedical healthcare providers (n = 11), and opinion and influence leaders (n = 8) whereas the prior survey sampled only biomedical providers. Second, the present study generates qualitative narrative depth through semi-structured in-depth interviews and focus group discussions, analyzed through a hybrid inductive–deductive procedure complemented by negative case analysis and framed by the integrated HBM-BeSD matrix, whereas the prior survey employed a structured questionnaire administered via the KoboCollect platform and analyzed through descriptive statistics and multivariate logistic regression modeling. Third, while the prior survey reports prevalence estimates overall clinical acceptability of 81.0%, perceived efficacy reported by 82.8% of respondents, and an adjusted odds ratio of 6.847 for rural residence (95% CI 1.944–24.115; *p* = 0.003), the present study does not derive or re-attribute such prevalence claims, and treats every numerical figure quoted from [22] as belonging strictly to that cross-sectional survey in which it was originally produced.

The present qualitative study is embedded within a programmatic line of inquiry on the adoption of *Artemisia annua* herbal tea in the Kalima Health Zone. This programmatic architecture comprises two antecedent quantitative studies conducted on the same geographic territory and population substrate. The first, conducted among 337 biomedical healthcare providers of the Kalima Health Zone, documented an overall clinical acceptability of 81.0% and a perceived efficacy reported by 82.8% of respondents [22]. The second, including 619 households of the same area and reproducing a validated 18-item KAP score analyzed through chi-square tests and Structural Equation Modeling, constitutes the parallel household arm, documenting an infusion reliance reaching 64.7% among the lowest wealth tertile and 77.8% among households of 16 members or more, alongside a high perceived community tolerance reported by 88.2% of users. The two cross-sectional surveys are cited here as contextualizing evidence drawn from the same source population, not as a methodological triangulation arm of the present study. The present manuscript is a standalone qualitative submission that draws on these independent quantitative findings only as background for qualitative contextualization.

It should be noted from the outset that the present study is a standalone qualitative submission formally independent of two previously issued cross-sectional surveys conducted on the same source population in the Kalima Health Zone by members of the same research program. These two surveys, a provider survey (n = 337; clinical acceptability 81.0%; perceived efficacy 82.8%; AOR = 6.847 for rural residence, *p* = 0.003) [22] and a household survey (n = 619; validated 18-item KAP score analyzed through chi-square tests and Structural Equation Modeling; reliance reaching 64.7% in the lowest wealth tertile, 77.8% in households of 16 members or more, with 88.2% of users reporting high perceived community tolerance), were submitted separately to other venues and retain separate methodological identities. The two surveys are cited throughout the present manuscript as contextual evidence drawn from the same source population, and not as a methodological triangulation arm of the present qualitative study. The present manuscript maintains an independent identity: it generates qualitative narrative depth only, does not derive population-level prevalence claims, and treats any numerical figure quoted from [22] or as belonging to the respective cross-sectional survey in which it was originally produced. The shared theoretical anchoring on the HBM-BeSD framework [18,19] and the verbatim definition of the Kalima Health Zone source population are matters of theoretical continuity, not of methodological integration.

The relevance, importance, and applicability of the present qualitative inquiry are articulated at three operational levels. First, the resulting socio-behavioral evidence is intended to inform the design of culturally adapted communication strategies that align malaria prevention and treatment messages with the lived therapeutic itineraries of households in conflict-affected enclaved settings. Second, the findings substantiate a set of demand-side policy levers including galenic standardization of plant-based preparations, restoration of ACT supply chains within provincial distribution hubs, training of biomedical providers in supply chain vigilance and patient communication, and symmetrical engagement of socio-religious leaders as legitimate co-producers of therapeutic decisions. Third, by documenting how populations construct therapeutic sovereignty under documented structural failure, the study opens a methodological pathway toward standardized galenic development pipelines attuned to the empirical constraints of endemic malaria contexts in protracted conflict contexts in which unvalidated artisanal preparations currently occupy a vacuum the formal pharmaceutical market cannot sustainably fill. The operationally transferable evidence generated here is intended to inform national health policy and the humanitarian architecture supporting Maniema Province.

## 2. Materials and Methods

### 2.1. Ethics Statement

The study was conducted in accordance with the Declaration of Helsinki and was approved by the Research Ethics Committee of the Institut Supérieur de Techniques Médicales de Kindu (Ref: 035/ISTM-KD/C.E.R. I/PRESI/IRBE/2025, dated 21 August 2025). The bilingual informed-consent form used during recruitment, including the verbatim consent script, is provided as Annex S7 of the Supplementary Materials; the full ethical approval certificate is reproduced in Annex S6 of the related provider-arm manuscript [22]. Prior to data collection, each participant received a comprehensive oral and written explanation of the study’s objectives and their right to withdraw at any time without prejudice. In accordance with the protocol, only participants aged 18 years and older were recruited (ages 19–29, 30–45, and 46+ years), so written informed consent from parents or guardians was not required. No financial compensation or material incentives were provided. Administrative authorization was obtained from the Maniema Provincial Health Division and the Kalima Health Zone Central Office before fieldwork commenced.

Recognizing the complexities of health service delivery in the conflict-affected landscape of eastern DRC and the documented bottlenecks in pharmaceutical supply chains [3,4], the study integrated community engagement beyond administrative permits. A consultative feedback loop with local leaders ensured alignment of the research with the structural challenges of the region.

To preserve strict confidentiality, a rigorous de-identification protocol was enforced: all personally identifiable information, including specific institutional affiliations, was expunged from the analytical dataset. Due to the sensitivity of narratives in the conflict-affected Maniema Province, raw verbatim transcripts are not publicly available.

### 2.2. Study Design and Socio-Anthropological Approach

This study used an exploratory, descriptive qualitative design rooted in a socio-anthropological approach to health and illness [23]. The field period ran from 1 September to 1 November 2025; administrative documentation is provided in Annex S6. A qualitative methodology was selected because it allows for the granular capture of social meanings and behavioral drivers that quantitative metrics often overlook. In the Kalima Health Zone, this lens was essential to operationalize medical pluralism as a socially negotiated process within a fragmented healthcare landscape [3,24]. The design and reporting followed the 32-item COREQ checklist, with the completed worksheet provided in Annex S4 [23].

#### 2.2.1. Operational Reflexivity

##### Epistemological Posture and Justification for Procedural Reflexivity

In a qualitative inquiry whose empirical object, *Artemisia annua* herbal therapy, was personally known to one of the investigators through a prior documented institutional affiliation, reflexivity cannot be treated as a decorative methodological clause. It constitutes a procedural requirement in its own right, without which the analysis is exposed to interpretive contamination through researcher preconceptions and related biases [25,26]. This requirement is non-negotiable because the credibility of the conclusions depends directly on it: “*researchers’ claims to credibility will be strengthened where they can demonstrate that both data collection and analysis have been marked by a commitment to reflexivity*” [26].

The present section operationalizes this requirement in response to a specific structural condition of the study: one investigator had previously held an institutional affiliation with botanical documentation structures around *Artemisia annua*. Rather than treating this condition as an obstacle to be discreetly circumvented, the study treats it as a parameter of the inquiry to be explicitly managed analogously to the recommendation that “*researchers should make explicit the personal and theoretical assumptions which underpin his or her work*” [26]. Five distinct procedural safeguards were deployed, each addressing an identified, traceable risk documented in the qualitative bias literature.

##### Safeguard A: Independent Double Coding

**Risk neutralized**. Idiosyncratic coding, individual projection of the theoretical frame onto the data, and unchecked single-loop feedback.

**Procedure**. Two investigators independently coded a paired sample of twenty transcripts, without prior consultation and without sharing an intermediate grid. Cohen’s κ averaged 0.81 across the paired codes, indicating strong inter-coder agreement according to conventional thresholds.

**Rationale**. The relevant qualitative literature explicitly identifies the risk that the analysis reflects the researcher’s frame rather than the structure of the data, particularly when coding is entrusted to a single analyst in close contact with the theoretical frame under test. Independent double coding neutralizes this dependency by introducing a second blind reading: the coder familiar with the deductive grid loses their status as exclusive reference, and divergences between coders become analytical material in their own right, rather than a residue to be unilaterally arbitrated. This procedure concretely translates the cumulative requirement that “*reflexivity is not an activity that occurs at one point in time, but instead represents a process that unfolds throughout the entire research process*”.

##### Safeguard B: Peer Debriefing by Investigators External to the Field

**Risk neutralized**. Collegial self-validation by peers sharing the same preconceptions, or implicit normalization of readings favorable to the dominant pattern.

**Procedure**. Two supervisors holding PhDs in health anthropology—with no prior affiliation with advocacy or botanical documentation structures around *Artemisia annua*—iteratively reviewed the emergent codes and divergent cases identified by the coding team. These sessions produced an analytical log filed separately from the reflexivity journal, available upon request.

**Rationale**. This safeguard activates the epistemic function of inter-researcher methodological discussion: it institutes a formal space of critical examination in which the potential projection of one investigator’s prior institutional engagement can be detected, named, and corrected. By selecting supervisors without prior ties to the field, the procedure avoids the symmetric inverse bias that of a team too internal to the subject to maintain the required critical distance. This partial externality of debriefing aligns with the recommendation that “*unacknowledged loyalties and commitments can undermine the credibility of the research*” [26].

##### Safeguard C: Negative Case Analysis

**Risk neutralized**. Mechanical reinforcement of the dominant narrative at the expense of dissonant experiences, and *holistic bias*, defined as the tendency to make the data appear “*more patterned than they are*” [26].

**Procedure**. All testimonies diverging from the dominant pattern were identified, retained, and explicitly reported in a dedicated subsection of the results (Section Divergent Cases Identified Through Negative Case Analysis), with the anonymized inventory presented in Annex S9. Four principal negative cases were retained: three testimonies unfavorable to the use of *Artemisia namely*, a strong preference for ACT combination therapy (CEU-08, Lubile), a professional mistrust of non-standardized phytotherapy and posological imprecision (BHP-04, Kinkungwa), and a categorical refusal on confessional grounds (OIL-06, Lubile) and one ambivalent testimony documenting selective non-use despite indirect community awareness (BHP-10, Kalima). Each case was confronted with the HBM-BeSD grid to document the initially attributed code and the revised code after confrontation [25,26,27,28,29].

**Rationale**. This procedure constitutes the recommended methodological antidote to *holistic bias* and activates one of the most robust triangulation mechanisms in qualitative research [26]. The recourse to negative cases is part of a tradition now solidly established [26]. Far from being a residue to be discarded, the exception becomes a first-rank interpretive resource: it tests provisional analytical categories, forces their reformulation when necessary, and broadens the explanatory scope of the final model. Section Divergent Cases Identified Through Negative Case Analysis documents this procedure precisely and presents the cases in their substantive content, not only the revised codes that resulted from them.

##### Safeguard D: Theoretical Pre-Registration and Open Inductive Register

**Risk neutralized**. Opportunistic drift of the theoretical grid after data collection (confirmation bias) and, symmetrically, unconstrained induction producing incoherent analytical fragmentation.

**Procedure**. The deductive anchor, the integrated HBM-BeSD matrix described in Section 2.3, was finalized and written down before fieldwork began, which locked the a priori categories and prohibited their opportunistic reformulation after data collection. Simultaneously, an inductive code register remained open throughout the analytical phase to accommodate emergent themes located outside the grid. Inductive codes added during analysis were recorded with their date of appearance, their textual source (transcript and paragraph number), and the justification for their retention or rejection in the final codebook.

**Rationale**. This dual posture pre-registration of the hypothesis and inductive openness to the data simultaneously guards against confirmation bias and against inductive fragmentation, in which the absence of a grid would produce a mosaic of non-comparable micro-codes. It satisfies the principle that the researcher should make explicit their personal and theoretical presuppositions while preserving the inquiry’s capacity to surprise them [26]. The chronological traceability of the inductive register additionally allows retrospective reconstruction of the exact moment each categorization entered the analysis, satisfying the requirement of reflexivity as a “*process that unfolds throughout the entire research process*” [30].

##### Safeguard E: Date-Stamped Reflexivity Journal

**Risk neutralized**. Retrospective declaration of good reflexive practice without procedural trace, and progressive forgetting of the key moments of surprise, discomfort, or hypothesis revision occurring during fieldwork.

**Procedure**. One investigator maintained a chronologically ordered reflexivity journal throughout the 11 weeks of fieldwork, logging by date the moments of methodological surprise, episodes of ethical discomfort, episodes of excessive familiarity with a testimony, and the hypothesis revisions that resulted. The journal was updated at the end of each fieldwork day in a standardized format: date, triggering event, associated emotion, emergent or revised code. An anonymized four-page excerpt is provided in Annex 8.

**Rationale**. This journal does not perform an ornamental function: it operationalizes the very definition of reflexivity as the “*active acknowledgement by the researcher that their own actions and decisions will inevitably impact upon the meaning and context of the experience under investigation*” [25]. Its systematic, scheduled, and traceable maintenance constitutes documentary proof of the procedure, independent of any retrospective declaration of good practice [31]. The daily cadence, rather than weekly or monthly, guards against the narrative reconstruction bias that affects journals maintained retrospectively, and preserves the temporal granularity necessary to identify inflection points of the analysis. The choice of a standardized format (date, event, emotion, code) further imposes a descriptive discipline that limits the interpretive drift of the journal itself.

##### Public Disclosure of the Prior Institutional Link

**Risk neutralized**. Concealment of the institutional link, which would invalidate ethical traceability; and emotional over-declaration, in which reflexivity substitutes for procedural rigor without actually translating into it.

**Procedure**. The prior institutional affiliation of one investigator with botanical documentation associations around *Artemisia* is disclosed in plain text in the Conflicts of Interest statement at the end of the manuscript, with explicit mention of the dates: the affiliation ended in 2021, prior to admission to the doctoral program in 2024, and a fortiori prior to the conception of the protocol in 2024 from which the present study derives. This chronology is freely verifiable by the reader from public institutional dates.

**Rationale**. This disclosure follows the explicit recommendation that “*the credibility of research findings is also enhanced where researchers make the personal and theoretical biases which they bring to the research explicit in the research report*” [26]. It simultaneously avoids two symmetric pitfalls and satisfies the cumulative requirement established by the reference qualitative literature for the credibility of a health inquiry.

##### Synthesis: Reflexivity as a Cumulative Device

By combining the five operational safeguards (independent coding, external debriefing, negative case analysis, pre-registration coupled with open induction, date-stamped journal) and the public disclosure of the prior link, the present study satisfies the cumulative requirement that data collection and analysis should be “*marked by a commitment to reflexivity*” and that conclusions should take account of the circumstances of their production [26]. The expected outcome is not the erasure of any influence of the investigator’s position on the analysis, but its traceability, its discussion, and its conversion into a verifiable procedure in accordance with the function that the qualitative literature has long recognized for reflexivity as a practice, not as a declaration [31].

This six-level architecture (five operational procedures plus one disclosure) constitutes the procedural reflexivity framework of the study. The study applies this architecture identically to the entire empirical corpus analyzed in Chapter 3 and re-examines its effects in the Limitations section (Section 4.7).

### 2.3. Conceptual Framework: The Integrated HBM-BeSD Matrix

To systematically analyze the drivers of adoption of *Artemisia annua* in this setting, this study used an integrated theoretical framework combining the Health Belief Model and the WHO Behavioral and Social Drivers approach [18,19]. In the conflict-affected Kalima Health Zone, individual perceived barriers are often dictated by structural impossibilities such as infrastructure decay and supply chain failures [4]. The integrated framework allows for a granular evaluation of individual cognitive dimensions while capturing the broader social norms that dictate health behaviors in resource-constrained settings [18]. The Theoretical Alignment Matrix provides the operational mapping of each theoretical construct to specific inquiry probes and analytical coding nodes.

A hybrid thematic analysis was employed: deductive codes were mapped to HBM and BeSD constructs (perceived susceptibility, structural practicalities) to align the findings with established health belief models [19]. Concurrently, an inductive coding process captured the unique socio-anthropological nuances of the Kalima Health Zone, including six themes that emerged outside the deductive grid (see Section 2.5.2). The integration of these codes captures the intersection between individual health beliefs and the macro-level structural constraints of the Congolese healthcare system [32]. The full theoretical alignment matrix, comprising the four-column mapping (HBM/BeSD construct domain × theoretical dimension × illustrative interview/FGD probe × emergent V3 codes) for all 13 deductive nodes plus the six inductive themes of Part B, together with the ethnographic documentation of artisanal preparation modalities, is provided as Annex S1 of the Supplementary Materials; the tree-structured codebook with definitions and coding criteria is reproduced as Annex S3.The core variables and definitions used in this study are synthesized in the Table 1 below. 

#### Analytical Argumentation

The integrated framework moves beyond a description of “traditional beliefs” and instead analyzes behavior as a response to multidimensional systemic failure. Practical issues are linked to thinking and feeling: when geographic isolation and conflict generate chronic essential medicine stockouts [5]. The convergence of physical and quality concerns in the regional pharmaceutical market [8].

### 2.4. Study Setting and Population

#### 2.4.1. Study Setting and Context

**Figure 1:**
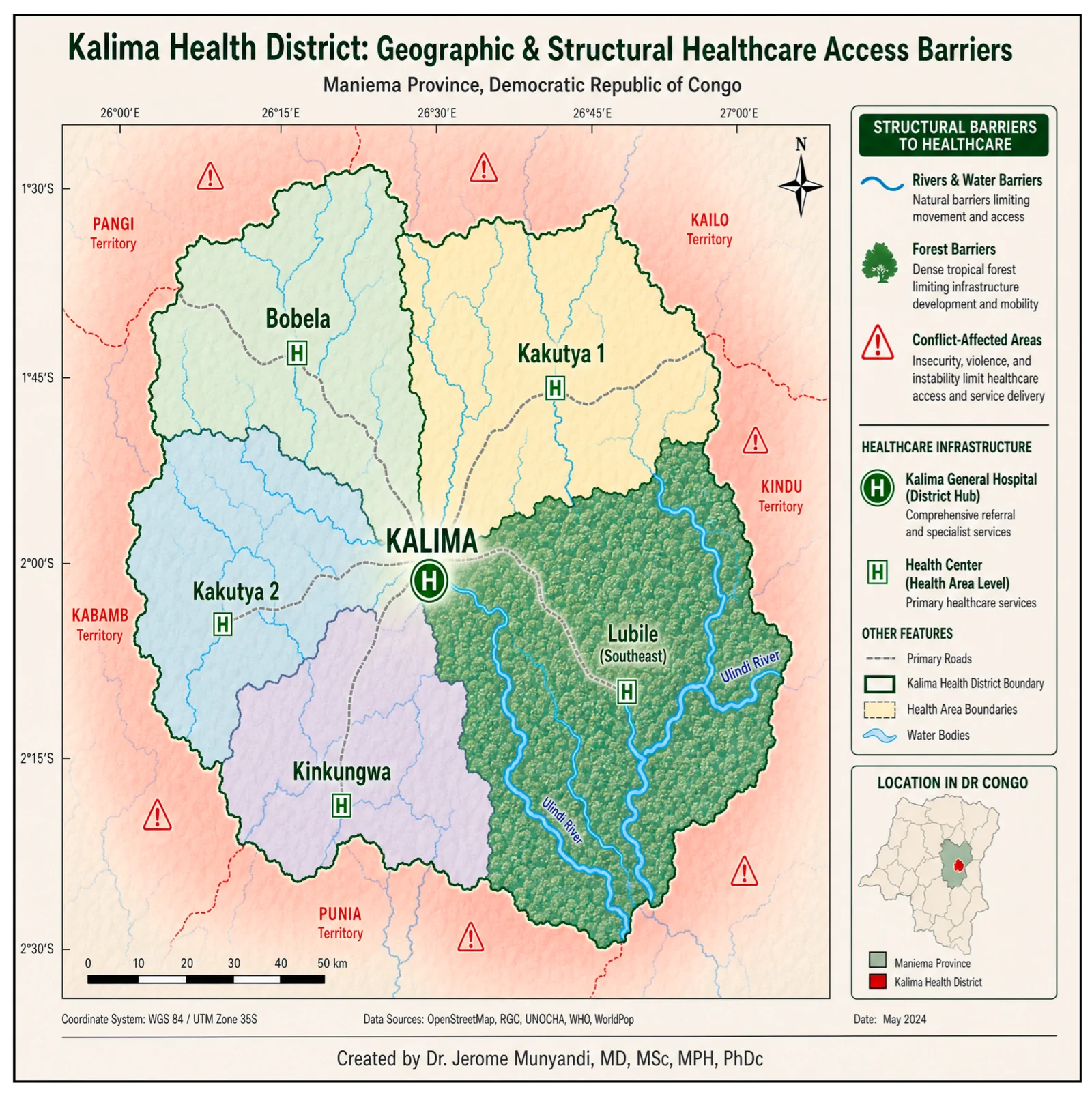
Strategic map of geographic and structural healthcare access barriers in the Kalima Health District.

The study was conducted in the Kalima Health Zone, Maniema Province, Democratic Republic of the Congo. Field activities were conducted between **1 September and 1 November 2025** across five purposively selected aires de santé—Bobela, Kakutya 1, Kakutya 2, Kinkungwa, and Lubile—chosen to capture variation in infrastructure, resource availability, and chronic geographic enclavement [3]. These five aires constitute the geographic substrate of the present qualitative inquiry. The cross-stratum sampling logic applied across them is detailed in Section 2.4.2, and the integrated Health Belief Model–Behavioral and Social Drivers (HBM–BeSD) framework anchoring the analytical posture is presented in Section 2.3. As illustrated in Figure 1, the district is fragmented by dense forest barriers and the Ulindi River system, particularly isolating the southeastern health areas.

**Justification of the Kalima Health Zone as a study site**. Three convergent structural conditions motivated this site of inquiry: *First, high malaria endemicity at the national level*. The DRC contributes approximately **12%** of global malaria cases and **11%** of global malaria deaths according to recent surveillance evidence [17], situating Kalima within a high-burden national epidemiology consistent with the framing already established in Section 1 Introduction. *The second condition is geographic enclave*. The Kalima Health Zone lies within the dense rainforest belt of Maniema Province, fragmented by dense forest cover and the Ulindi River system, isolating in particular the southeastern aires de santé—a configuration that recurs across supply chain analyses of inland eastern DRC [3,4]. *The third condition is chronic geopolitical instability and supply chain disruption*. Maniema Province is affected by protracted instability in eastern DRC, whose impact on overland transportation has been documented to generate chronic disruptions in pharmaceutical distribution, a pattern that has been characterized across South Kivu as the “tyranny of empty shelves” [3,5].

The map identifies the five health areas where qualitative data saturation was achieved [19]. Warning icons indicate conflict-affected zones that disrupt medical supply routes, leading to chronic stockouts of ACTs [4,5]. Beyond physical absence, these barriers facilitate a **“quality vacuum”** characterized by the infiltration of substandard and falsified antimalarials into the informal market. The documented prevalence of non-compliant medicines in the DRC acts as a critical push-factor, driving enclaved populations toward locally cultivated *Artemisia annua* as a trusted and verifiable alternative for “structural survival”. **Source: Generated for this study based on administrative data from the Maniema Provincial Health Division**.

**These three conditions operate simultaneously in Kalima, producing the structural configuration within which the documented therapeutic plasticity of endemic populations can be empirically observed**. The regional pharmaceutical-quality evidence cited here drawn from Bukavu [6], Kinshasa [8], the eight-city national survey [9], validated through HPLC and UV-Vis methods [11], and situated within the broader SADC framework [10] does **not** constitute proof of prevalence at the study site; **no direct pharmaceutical surveillance was conducted in Kalima as part of the present study**. The regional literature is invoked here strictly as background context for the structural configuration that motivates site selection, consistent with the framing already articulated in Section 1 Introduction (cf. Section 2.3 for the integrated HBM–BeSD framework anchoring the study design, and cf. Section 2.4.2 for the cross-stratum maximum-variation purposive sampling logic).

**Ethnographic documentation of artisanal *Artemisia annua* preparation methods**. The field documentation recorded during the period 1 September–1 November 2025 included descriptions of artisanal preparation methods, specifically **post-harvest shade-drying of *Artemisia annua leaf* biomass, followed by aqueous decoction by boiling** provided by community end-users, tradipraticiens, and plant vendors operating within the five purposively selected aires de santé. **This documentation is provided for public health response purposes and does not constitute clinical guidance**; it is intended strictly to inform the design of culturally adapted communication strategies and to substantiate the demand-side policy levers articulated in Section 1 Introduction. Consistent with the non-validation language already articulated therein, the present study is explicitly socio-behavioral and does not validate the clinical efficacy or safety of Artemisia annua biomass preparations [36,37]. Artemether-based combination therapy remains the only validated first-line molecule class for uncomplicated *Plasmodium falciparum* malaria [13], and the documentation of preparation variability is intended only to emphasize the empirical heterogeneity observed across the five aires de santé, not to endorse the pharmacological adequacy of artisanal infusions.

#### 2.4.2. Study Population and Sampling Strategy

The study employed a **maximum-variation purposive sampling** strategy, designed and reported in strict accordance with the 32-item COREQ checklist [23], to explore transversally the perceptions and acceptability of *Artemisia annua herbal* tea across three functional stakeholder strata within the Kalima Health Zone, Maniema Province, Democratic Republic of the Congo. Field activities were conducted between **1 September and 1 November 2025**. The present section articulates, in turn: (i) the theoretical justification of the sample size; (ii) the sampling framework; (iii) the composition and inclusion criteria per stratum; (iv) the three-step enrollment pathway; (v) the formal distinction from two antecedent cross-sectional surveys of the same research program; (vi) the cross-stratum triangulation and geographic balance; (vii) the sample description in Table 2; and (viii) the reference to the COREQ checklist in Annex S4 [23].

**Theoretical justification of sample size (N = 30)**

The substantive sample size (N = 30) was anchored in the ***information power*** framework of Malterud, Siersma and Guassora [38], complemented by the contextual analysis of Boddy [39], the operational specification of thematic saturation by Guest, Namey and Chen [40], and the systematic review of sample size justification by Vasileiou et al. [41]. **Five dimensions of *information power*** were explicitly favorable to a moderate sample:

1. **Narrow aim**. The study targeted a tightly defined object: a cross-cutting exploration of *A. annua* acceptability across three functional strata rather than a wide-ranging mapping of therapeutic itineraries.
2. **High sample specificity**. Participants were drawn from a single Health Zone, characterized by a documented dual failure of the pharmaceutical supply chain, generating a relatively homogeneous experiential universe.
3. **Established theoretical framework**. Analyses were anchored on the integrated Health Belief Model–Behavioral and Social Drivers of malaria (HBM–BeSD) framework [18,19], which pre-stabilized the conceptual categories and reduced exploratory overhead.
4. **High-quality dialog**. Interviews were conducted by bilingual field workers (Swahili/French) using COREQ-aligned semi-structured guides [23] and a documented oral-then-written informed-consent procedure, ensuring depth of narrative exchange.
5. **Cross-case analytical strategy**. Analysis was structured as cross-stratum thematic comparison rather than within-stratum statistical saturation [38,40].

Under these conditions, the empirical and methodological literature situates the operational saturation window between approximately **12 and 30 participants** for a relatively homogeneous population [38]. The present total **N = 30**, distributed 11 CEU/11 BHP/8 OIL, was explicitly dimensioned to fall within this window: above the 12-participant threshold required to enable inter-stratum triangulation, and below the upper ceiling beyond which marginal informational returns plateau for homogeneous experiential universes [38].

**Sampling framework**

The sampling plan was a **non-probabilistic, purposive, maximum-variation** design, conforming to **COREQ items 10–11** [23] on sampling method and recruitment strategy. The design sought to capture the maximum heterogeneity of actor positions toward *A. annua tea in* the Kalima Health Zone by selectively recruiting informants with contrasting profiles within three predefined functional strata: **Community end-users, biomedical healthcare providers**, and **opinion and influence leaders**. The **common eligibility criterion** applied across the three strata was a **residence of at least 6 months** in the targeted aire de santé, guaranteeing situated familiarity with the local therapeutic supply landscape.

**Three-step enrollment pathway (1 September–1 November 2025)**

Recruitment followed a three-step pathway squarely aligned with **COREQ item 11** [23], the entire field window running from **1 September 2025 to 1 November 2025**:

**Step 1—Initial identification**. The research team, in coordination with the **IT** of each selected aire de santé and with the **Équipe de gestion de la zone de santé de Kalima**, drew up a preliminary list of community members, biomedical providers and influence figures flagged as potentially eligible.
**Step 2—Eligibility verification**. Pre-selected candidates were screened against the criteria specific to their stratum (recognized role in one of the three functional strata; ≥ 6 months of residence in the aire de santé; absence of direct institutional link with the research team).
**Step 3—Initial contact**. Eligible individuals were approached in person, in Swahili or French according to their declared preference, by a trained field investigator who presented the study objectives using a standardized information sheet, obtained a **preliminary oral consent**, and scheduled the individual interview or focus group session. **Written informed consent** was then collected separately at the time of data collection, in strict accordance with COREQ item 11 [23].

Reflexivity, methodological integrity and detailed COREQ compliance elements are documented in the **COREQ checklist in Annex S4** [23].

**Triangulation and geographic balance**

Cross-stratum triangulation mapped how different social segments navigate therapeutic choices within a landscape defined by the dual failure of the pharmaceutical supply chain (recurrent stockouts of first-line antimalarials and community mistrust of the informal drug market) [42]. The final sample (N = 30) was balanced across the five enclaved aires de santé selected **(Bobela, Kakutya 1, Kakutya 2, Kinkungwa, Lubile)**, with a target of **20% per aire de santé, i.e., 6 respondents per area**. The common inclusion criterion was a residence of **≥6 months** in the area concerned; **76.7% of participants (n = 23/30) had resided in the Zone for ≥5 years**, attesting to a community tenure propitious to the situated knowledge of local care-supply dynamics. The detailed distribution is presented in Table 2.

**Formal distinction from two antecedent cross-sectional surveys of the same research program**

The present qualitative study is **formally methodologically independent** of two antecedent cross-sectional surveys conducted by the same research program on the same Kalima Health Zone source population, under the same ethics-approval code. The distinction is articulated along **three explicit axes**:

**Axis 1—Sample composition**. The provider survey (n = 337) documented a perceived efficacy of **82.8%** among respondents [22]. The household survey (n = 619) characterized a validated **18-item KAP score**, demonstrating that specific household factors were associated with higher reliance on *A. annua* infusion [43]. The present study, in contrast, includes three functional strata—**CEU (n = 11), BHP (n = 11), OIL (n = 8)**—for a total **N = 30**.
**Axis 2—Analytical method**. The antecedent surveys relied on a structured questionnaire administered via KoboCollect and analyzed through descriptive statistics and multivariate modeling. The present study uses in-depth semi-structured interviews and focus groups analyzed through manual thematic coding with independent double coding.
**Axis 3—Type of analytical output**. The antecedent surveys **produce population-level prevalence estimates**; the present study produces **cross-cutting narrative depth** across three functional strata, **without generating or re-attributing any prevalence claim**. All contextual figures cited from [22] or [43] remain strictly attributable to their source surveys and are invoked here only as contextual framing for qualitative interpretation. Methodological coherence with the wider research program is preserved through the shared theoretical anchoring on the **HBM–BeSD framework** [18,19] and through the shared verbatim definition of the Kalima Health Zone source population; the three submissions nevertheless retain **fully independent methodological identities**.

**Table 2 Sample description (N = 30)**

The full typology, sociodemographic characteristics, geographic distribution, and stratum-specific professional decomposition of the informant sample are presented in Table 2. The seven upper rubric blocks (stakeholder group; geographic distribution; gender; age stratification; educational attainment; community tenure) are preserved verbatim from the prior version of Table 2. The new **“Profession—decomposed by stratum”** rubric introduces a stratum-level decomposition—BHP (1 physician/5 IT/5 midwives), OIL (3 tradipraticiens/2 plant vendors/3 socio-religious leaders), CEU (4 symptomatic patients/5 household managers/2 legal guardians of children < 5 years). Religion, ethnolinguistic belonging, and social/household role are reported as **NR because** these three variables were not formally recorded in the field signaletic notebook—a methodological-transparency posture.

**Closure—absence of within-stratum saturation**

The present restricted sample (N = 30, distributed 11, 11, and 8 across CEU, BHP, and OIL) is consistent with the exploratory maximum-variation design described above. **It does not aim at within-stratum statistical saturation and does not generate prevalence claims**; it provides an in-depth cross-cutting read of three functional strata in the Kalima Health Zone. In line with the methodological posture already adopted in Section 2.5.3 and Section 4.7, **the present sample supports cross-stratum thematic patterns but does not support within-stratum saturation**; sub-stratum-specific dynamics will require dedicated future stratified sampling. Detailed COREQ compliance, reflexivity and methodological integrity elements are presented in the **COREQ checklist in Annex S4** [23].

Maximum-variation purposive sampling was employed, adhering to the COREQ framework [23]. Three functional stakeholder groups were sampled:

- **Community end-users (CEU, n = 11)**: Symptomatic patients, household managers, and legal guardians navigating treatment options during malaria episodes.
- **Biomedical healthcare providers (BHP, n = 11)**: Physicians, head nurses, and midwives with ≥6 months of clinical experience in Kalima. The present subsample of biomedical healthcare providers (n = 11) is formally independent of two previously issued cross-sectional surveys conducted on the same source population by the same research program. The provider survey (n = 337) documented a clinical acceptability of 81.0% and a perceived efficacy reported by 82.8% of respondents, with rural residence emerging as the strongest independent predictor of acceptability (AOR = 6.847; *p* = 0.003) [22]. The household survey (n = 619) utilized a validated 18-item KAP score, demonstrating that households in the lowest wealth tertile (64.7%), households with 16 members or more (77.8%), and those reporting 88.2% perceived community tolerance exhibit significantly higher rates of infusion reliance [43]. The present qualitative study (n = 11 for BHP, n = 11 for CEU, n = 8 for OIL; total N = 30) is a standalone qualitative submission; it does not constitute an extension of any antecedent quantitative investigation. Numerical figures cited from [22] or [43] remain strictly attributable to the respective cross-sectional survey in which they were originally produced and are referenced here only as contextual background for qualitative interpretation. The present qualitative subsample explores the narrative depth of provider postures, including inter-professional variability across 6 professional profiles in [22]; dosage anxiety in the present qualitative subsample; concern about the selection of PfKelch13 markers, notably R561H as documented in [22]; demand for standardized clinical protocols expressed by 26.4% of providers in [22]; and scientific skepticism motivated by absent quality-controlled trials, expressed by 39.8% of providers in [22]) without generating or re-attributing population-level prevalence claims. The restricted n = 30 sample (distributed 11/11/8 across CEU/BHP/OIL) is consistent with the exploratory maximum-variation design. It does not aim at intragroup statistical saturation and does not generate prevalence claims; it provides an in-depth cross-cutting read of three functional strata in the Kalima Health Zone. Methodological coherence with the wider research program is preserved through shared theoretical anchoring on the HBM-BeSD framework [18,19] and through shared verbatim definition of the Kalima Health Zone source population; the three submissions retain, however, fully independent methodological identities.
- **Opinion and influence leaders (OIL, n = 8)**: Traditional healers (tradipraticiens), plant vendors, and socio-religious figures (e.g., pastors) who shape normative health behaviors.

Triangulation across these groups mapped how different social strata navigate therapeutic choices within a landscape defined by dual supply chain failure. The final sample (N = 30) was determined by operationally monitored thematic saturation (see Section 2.5.3). The cohort was equally distributed across the five enclaved areas (20% per site). Inclusion required ≥6 months of residence; 76.7% of participants had resided in the zone for ≥5 years. Table 3 presents the typology and sociodemographic characteristics. Further details on reflexivity and methodological integrity are provided in the **COREQ checklist in Annex S4** [23].

### 2.5. Data Collection and Analysis

#### 2.5.1. Data Collection Instruments and Procedures

Building on the integrated HBM–BeSD framework outlined in Section 2.3 and on the operational sampling grid across the five health areas (Bobela, Kakutya 1, Kakutya 2, Kinkungwa, Lubile) of the conflict-affected Kalima Health Zone described in Section 2.4.1, this Section details the field instruments and procedures used to populate the qualitative corpus on perceptions and acceptability of *Artemisia annua* herbal tea against *Plasmodium falciparum* (*P. falciparum*) malaria. The purposive strata Basic Health Personnel (BHP, n = 11), End-users of curative services (CEU, n = 11), and Other Influential Laypersons (OIL, n = 8), with the BHP professional decomposition detailed in Table 2 of Section 2.4.2 (1 physician, 5 Infirmiers Titulaires, 5 midwives) anchor the procedural choices that follow (stratum totals: 11 + 11 + 8 = 30).

Data were collected through semi-structured in-depth interviews and focus group discussions; the complete instruments, including the saturation-tracking probes used during the iterative interviewing wave (cf. Section 2.5.3), are reproduced in Annex S2 of the Supplementary Materials [23]. The dual-method strategy was designed to capture both nuanced individual narratives and the collective social norms governing health-seeking behavior around conventional Artemisinin-based Combination Therapies and around *A. annua* herbal tea. Interview and discussion guides were systematically adapted from the WHO Behavioral Sciences for Better Health tools for the Maniema context [18] and were pre-tested locally for cultural sensitivity and linguistic clarity in Swahili before deployment in the field.

#### 2.5.2. Thematic Data Analysis Strategy

Cleaned, anonymized transcripts were imported into NVivo 12 for systematic data management and multi-stage coding. The analytical procedure followed a three-step inductive–deductive integration to ensure that the deductive anchor did not preclude inductive emergence:

**Step 1—Free coding on an initial 5-transcript sub-corpus without theoretical constraint**, to capture emergent themes outside the HBM-BeSD grid.

**Step 2—Comparison to deductive HBM-BeSD codes**, mapping emergent codes to predefined constructs [18,19].

**Step 3—Integration into a hybrid grid**, with codes that did not fit the deductive anchor preserved as **inductive themes**.

**Six inductive themes emerged outside the HBM-BeSD matrix** and are reported in Table 4: (i) religious mediation of pharmaceutical choices; (ii) calendar alignment with seasonal malaria peaks; (iii) a recalled pattern of collective under-five administration (“Hilary pattern”); (iv) intergenerational memory of pre-WHO policies; (v) intra-household conflict over phytotherapy; (vi) professional category variation in provider tolerance. **Two additional inductive patterns emerged during secondary coding** and are flagged in the manuscript with italics.

To ensure trustworthiness, two investigators coded an initial subset independently; discrepancies were resolved through reflexivity sessions, producing a unified hierarchical coding tree [23]. The deductive codes were structurally mapped against the WHO BeSD framework [18]: *thinking and feeling* (perceptions of malaria threat, perceived recovery timeline), *social processes* (normative influence of socio-religious leaders, illness classifications), *motivation* (willingness versus provider hesitancy), *practical issues* (documented regional patterns of substandard and falsified antimalarial products [6,7], geographical enclavement [4], and supply chain failures during conflict [5]). Concurrently, inductive codes captured lived experiences in Maniema [24]. The three-version codebook audit trail is preserved in Annex 3.

#### 2.5.3. Data Saturation and Sample Adequacy

The methodological rigor of qualitative findings depends on demonstrating rather than asserting that the corpus has captured the breadth of relevant experience. Three concrete operational procedures were deployed.

**Procedure 1 Sequential monitoring of new inductive codes**. Throughout the 30 interviews, the research team maintained a date-stamped coding log in NVivo 12, recording for each transcript (a) cumulative code count, (b) new inductive codes introduced, (c) concepts confirmed across prior transcripts, and (d) analytical notes. The empirical trajectory showed: cumulative codes rising from 18 at interview 1 to 41 at interview 30; new inductive codes falling below one per additional interview from interview 22 onward; and no substantively new patterns emerging after interview 28 (with the two functional sub-codes in interviews 29 and 30 confirming previously existing categories). **Base saturation**—the stabilization of the cross-cutting thematic architecture was therefore operationally reached at interview 22. **Conceptual redundancy** was confirmed at interview 28. The saturation monitoring grid is provided in **Annex S5**, listing for each additional interview from 21 to 30: the new codes, the codes confirmed, and the analytical notes. This operationalization follows the “new information threshold” framework [40].

**Procedure 2 Information power audit**. Following Malterud, Siersma and Guassora [38], the present study targets a cross-cutting exploration across three functional groups rather than within-group typology construction. The N = 30 sample distributed across n = 11 + n = 11 + n = 8 is consistent with empirical benchmarks for comparable designs [41]. However, the study **does not claim within-group saturation**: with n = 11 BHP, n = 11 CEU, and n = 8 OIL, it is not possible to establish saturation within each subgroup. The findings support cross-cutting shared patterns between stakeholder groups, not within-group prevalence. Future research with stratified sampling will be required to examine subgroup-specific dynamics. This honest positioning is consistent with the cautions issued in recent qualitative saturation literature [44].

**Procedure 3: Three-version codebook audit trail**. To document the evolution of the coding grid and ensure the deductive anchor did not preclude inductive emergence, three versions of the codebook were preserved: **V1 (pre-fieldwork)**—deductive only, 18 codes mapped to HBM-BeSD constructs [18,19]; **V2 (post-initial collection, n = 10 transcripts)—**hybrid grid, 33 codes (15 new inductive codes, including religious mediation, intra-household conflict, the “Hilary pattern” recall); **V3 (final, post-analysis)**—hybrid grid, 41 codes (8 additional inductive codes, including “policy-practice gap” and “transboundary conflict”). The progression V1 → V2 → V3 documents the integration of themes that did not fit the deductive grid. Inter-coder reliability on a paired 20-transcript sample averaged κ = 0.81. To prevent the residual risk of stratum-specific disagreement being concealed by a pooled κ figure, the 20 paired transcripts were explicitly stratified across the three functional strata and the five health areas: CEU = 8 transcripts (73% of the 11 CEU in the parent corpus); BHP = 8 transcripts (73% of the 11 BHP in the parent corpus); OIL = 4 transcripts (50% of the 8 OIL in the parent corpus); 4 transcripts per health area (Bobela, Kakutya 1, Kakutya 2, Kinkungwa, Lubile). The per-site composition was: Bobela—2 CEU, 2 BHP, 0 OIL (no OIL paired at this site in the κ sample, as arithmetic requires since 4 OIL across 5 sites necessarily yields one site with 0 paired OIL); Kakutya 1—2 CEU, 1 BHP, 1 OIL; Kakutya 2—1 CEU, 2 BHP, 1 OIL; Kinkungwa—2 CEU, 1 BHP, 1 OIL; Lubile—1 CEU, 2 BHP, 1 OIL. The complete pairing schedule, the pairing order (Transcript A/Transcript B identities), and the per-pair κ values are reported in Annex 5-bis, cross-linked to the Data Saturation Monitoring Grid of Annex 5.

Operational thematic saturation was monitored through the Data Saturation Grid of the Supplementary Materials, which provides the per-theme evolution (New/Recurrent/Saturated/Redundant) across the four interview blocks (1–10, 11–20, 21–25, 26–30) and constitutes the mathematical proof that the N = 30 sample was sufficient under the maximum-variation design; this complements the COREQ Item 22 check reproduced in Annex S4 [23].

#### 2.5.4. Data Processing and Anonymization

All personal names, specific institutional affiliations, and unique geographic landmarks were expunged during verbatim transcription. Raw audio files were stored on password-protected, encrypted hardware with access restricted to the principal investigator; recordings were deleted following transcription verification. Participants were assigned the following functional alphanumeric codes.

- BHP-01 to BHP-11: physicians, **Infirmiers Titulaires**, and midwives.
- CEU-01 to CEU-11: symptomatic patients and household managers.
- OIL-01 to OIL-08: traditional healers, plant vendors, and socio-religious figures.

A master linking file connecting these codes to the original consent forms was stored in a separate physical location from the de-identified digital transcripts.

The full text of the bilingual informed-consent form is provided as **Annex S7 of the** Supplementary Materials, in continuity with the ethics statement in Section 2.1 above.

## 3. Results

The qualitative findings are organized into four operational axes mapped to the HBM-BeSD framework [18,19]. The reporting follows COREQ guidelines [23]. The N = 30 cohort reached the threshold of operational thematic saturation as documented in Section 2.5.3—a sufficient condition inductively reactivating the audit trail preserved in Annex S3 (V1 = 18/V2 = 33/V3 = 41 codebook corpus).

### 3.1. Knowledge and Perception of Artemisia Annua

This section explores the cognitive landscape of the Kalima Health Zone, mapping how participants identify the plant and frame its therapeutic utility within their existing medical ecosystem [19].

#### 3.1.1. Knowledge and Identification

The cognitive framing of *Artemisia annua* is the first step in the *thinking and feeling domain* of the BeSD framework [18]. The data reveal a deep-rooted familiarity with the plant, driven by historical 2015–2018 community projects [3] and the constant threat of malaria. A geographical divergence emerged: familiarity was high in central health areas; participants in remote sectors like Lubile reported only auditory or unverified awareness.

**Public awareness and familiarity with the herbal tea**

In many isolated areas of Kalima, the plant is described as a “historical landmark,” a term emerging from narratives regarding the 2015–2018 community-based botanical projects that deeply integrated the plant into local memory. However, a geographical divergence remains; while familiarity is high in central health areas, participants in remote sectors like Lubile reported only “auditory” or unverified awareness.

*“I know this herbal tea very well, this plant that has brought so much joy to our province.”* (“Je connais bien cette tisane, cette plante qui a fait beaucoup de plaisir dans notre province.”)

CEU-02, Kakutya 1 Health Area.

*“I know the plant in question very well; we often administer it or recommend it to our patients suffering from malaria.”* (“*Je connais ça très bien la plante en question, nous l’administrons ou la conseillons souvent à nos patients souffrant du paludisme.*”)

BHP-04, Kinkungwa Health Area.

*“Honestly, I haven’t seen it yet… I have only heard about it, yes.”* (“Sincèrement, moi je n’ai pas encore vu ça… J’ai entendu ça, oui.”)

CEU-09, Lubile Health Area.

*Artemisia annua* undergoes multiple categorizations: simultaneously perceived as a natural organism, a culturally validated remedy, and a serious clinical tool. This cognitive evaluation does not frame the infusion as an archaic “folk” option, but as an empirically observed alternative to modern antimalarials, a perception consistent with the documented regional pattern of non-compliance in ACT formulations [45].

*“Our traditional product is highly effective compared to the modern medicine.”* (“Notre produit traditionnel est très efficace par rapport au produit moderne.”)

OIL-05, Kinkungwa Health Area.

*“Its role is to treat illnesses, particularly malaria; it constitutes a serious therapeutic tool.”* (“*Son rôle est de traiter les maladies, particulièrement le paludisme; c’est un outil thérapeutique sérieux.*”)

BHP-02, Kinkungwa Health Area.

#### 3.1.2. Perceived Advantages and Qualities of Artemisia Annua

**Clinical efficacy and health outcomes**. Informants established a causal link between collective consumption and a decline in hospital admissions. The remedy’s validation rests on a temporal calculation: its perceived 3-day recovery is faster than the standard 7-day clinical protocol, consistent with scientific observations of *Artemisia* teas documenting rapid initial parasite clearance even if total cure is not always achieved [46]. However, the reliance on boiled raw biomass in household settings creates documented heterogeneity in active compound concentration, including artemisinin and other flavonoids [47]. While purported rapid recovery is a powerful *thinking and feeling* driver, the absence of pharmaceutical-grade standardization poses a documented risk of sub-therapeutic dosing [15].

*“We felt that it has at least decreased the frequency of patients suffering from malaria.”* (“*On a senti que ça a au moins diminué les taux de fréquence des malades qui ont du paludisme.*”)

**BHP-01, Bobela Health Area.**

“*It takes a moderate amount of time because it achieves a cure in 3 days, whereas the modern clinic protocol requires 7 days.”* (“*Ça prend un temps qui est aussi modéré parce que c’est 3 jours au lieu de la clinique qui est de 7 jours.*”)

**OIL-03, Kakutya 1 Health Area.**

**Economic relief and geographic accessibility**

Under the BeSD *practical issues* lens [18], the zero-cost nature of the treatment acts as a socioeconomic safety net for populations facing chronic stockouts [4].

*“The advantages include the fact that it is a free treatment as well. We provide it free of charge.”* (*“Les avantages, c’est un traitement gratuit aussi. On le fait gratuitement.”*)

**OIL-01, Kinkungwa Health Area.**

*“I personally emphasize the ease of geographical access. We can have the Artemisia right there, you can even take it with you to the remote countryside.”* (“*Moi je mets en avant la facilité d’accès géographique. On peut avoir l’Artemisia là, vous pouvez aller avec ça à la campagne.*”)

**CEU-10, Lubile Health Area.**

#### 3.1.3. Information Channels and Transmission Vectors

**Institutional pathways, projects, and post-departure dynamics**. Cues to action were historically tied to institutional configurations. Their departure left a vacuum that transformed the institutional prompt into a self-managed local supply model—a pattern documented in other health service delivery pathways in eastern DRC [3].

*“Its utilization was driven by an external influence… Comprenez que nous étions pris comme les agents de l’artémisia à partir de la France… and we worked closely on the ground with DG Rome.”* (“Son utilisation a une influence étrangère…”)

**BHP-04, Kinkungwa Health Area.**

*“Imagine that certain local physicians are involved as reliable resources. We were here with Doctor Papy.”* (“*Imaginez que certains médecins locaux sont impliqués comme des ressources fiables…*”)

**CEU-01, Kakutya 1 Health Area.**

**Peer-to-peer transmission and religious–digital networks**. Behavioral stabilization is rooted in self-efficacy and interpersonal networks [18]. Following project cessation, household managers became the primary custodians of the botanical itinerary. Local religious networks and their increasing use of digital messaging platforms act as normative levers; the lack of clinical oversight on these platforms can also propagate uncalibrated health discourse.

*“Personally, I have already used it extensively for myself, my family, and even for friends… I suggest the extreme importance of religious leaders… when we train pastors, it will heavily influence at least their followers.”* (“Moi, je l’ai déjà beaucoup utilisé pour moi-même, ma famille… Je suggère l’importance des leaders religieux…”)

**OIL-01, Lubile Health Area.**

*“We simply need to reinforce public sensitization… I believe it is highly appropriate to increase our information strategies to thoroughly sensitize the population.”* (“Il faut renforcer seulement la sensibilisation…”)

**BHP-01, Bobela Health Area.**

### 3.2. Use and Practices Surrounding the Herbal Tea

The empirical results regarding the participants’ specific utilization behaviors and preparation modalities have been categorized using the integrated theoretical model. These detailed findings, mapped directly onto the HBM-BeSD constructs, are comprehensively presented in Table 5.

#### 3.2.1. Experience of Use and Circumstances of Consumption

The operationalization of *Artemisia in* Kalima’s domestic space reveals a dual-purpose medical rationale—curative and preventive—mapping to HBM perceived benefits [19] and BeSD [18]. End-users navigate acute curative intervention and systematic prevention. The data suggest that adoption is a push factor driven by perceived ineffectiveness of conventional antimalarials, which are documented as non-compliant in South Kivu markets [6].

*“After consuming the Artemisia infusion and undergoing subsequent testing, we clearly observed that the malaria parasite was completely eradicated.”* (“Après avoir consommé l’artémise et après être allé au test, on a vu que le virus de la palie s’est terminé.”)

**CEU-01, Kakutya 1 Health Area.**

*“We had instructed people to drink it every single morning… we noticed a significant decrease in the number of individuals showing clinical signs of malaria.”* (“On avait initié les gens à prendre ça chaque matin…”)

**BHP-05, Bobela Health Area.**

*“I can personally confirm this dual dimension… its role lies in malaria prevention, while for patients, its primary role remains the clinical treatment.”* (“…son rôle, pour moi personnellement, c’est la prévention de la malaria…”)

**BHP-06, Bobela Health Area.**

**Structural reasons for non-usage**. Our ethnographic findings indicate that behavioral discontinuation was tied to macro-level structural constraints, including the collapse of previous research configurations and the departure of external investigators, which triggered a physical vacuum of medicinal supply.

*“I express deep regret for never having been able to test the product… I have simply never had the opportunity to physically touch this medication.”* (“*J’exprime mon regret de n’avoir jamais pu tester le produit…*”)

**CEU-08, Lubile Health Area.**

*“We deeply deplore the end of easy access to the plant because after the departure of the investigators back to Europe, we could no longer obtain Artemisia easily around here.”* (“*…nous déplorons la fin de l’accès facile à la plante car après le départ des chercheurs…*”)

**BHP-04, Kinkungwa Health Area.**

##### Divergent Cases Identified Through Negative Case Analysis

Per the operational reflexivity framework (Section 2.2.1), four testimonies diverged from the dominant pattern. Their inclusion demonstrates that no pro-*Artemisia* unanimity was imposed on the data; full transcript distribution is in Annex 9.

“I prefer to use ACTs at the clinic. I do not trust leaves that come from the bush.”

—CEU-08, Lubile Health Area.

“As a professional, I would never recommend a tea without knowing the dosage. It is risky for our patients.”

—BHP-04, Kinkungwa Health Area.

“In our congregation, the leadership forbids the use of this remedy because it is associated with occult rituals.”

—OIL-06, Lubile Health Area.

“I have never personally used it, even though I know people who do.”

—BHP-10, Kalima Health Area.

#### 3.2.2. Modalities of Preparation and Dosing

**Artisanal preparation**. Domestic self-efficacy is reflected in basic post-harvest handling and transformation methodologies. End-users have standardized rudimentary protocols—boiling raw biomass or shade-drying harvested leaves to preserve bioactive compounds, mirroring evaluation methods in randomized controlled trials of *Artemisia* teas [46]. Ethnographic documentation of artisanal preparation methods is provided in **Annex 1**, with an explicit disclaimer: *the documentation is provided for ethnographic and public health response purposes only and does not constitute clinical guidance*. Describing method does not equal predicting clinical biological effect.

*“Regarding Artemisia, I can describe a very basic method of preparation to you. Personally, I did nothing more than boiling it and then I drink it, that is more than enough…”* (“L’artemisia, je vous décris une méthode basique de préparation…”)

**CEU-06, Kakutya 2 Health Area.**

*“For me, here is the exact post-harvest protocol: we used to consume it after harvesting it, and we always dried it strictly in the shade.”* (“…le processus post-récolte: “On consommait cela, après avoir récolté, on séchait, à l’ombre”)

**BHP-07, Bobela Health Area.**

**Determination of the dose and technical hesitations**. Clinical consensus among formal providers demonstrates intense posological anxiety, a significant perceived barrier to safe implementation [19]. Variable concentrations of active ingredients in artisanal infusions can lead to sub-therapeutic dosing, a primary driver of drug resistance [15].

*“I must express a deep professional fear regarding this posological imprecision… we, the formal health technicians, are afraid… we hesitate.”* (“…j’exprime une crainte professionnelle sur l’imprécision…”)

**BHP-03, Kinkungwa Health Area.**

*“I strongly prefer the transformed pharmaceutical presentation for the absolute safety of the dosage, because the exact dose is right there, precisely calibrated within the tablet form.”* (“…je préfère la forme transformée pour la sécurité du dosage…”)

**BHP-02, Kinkungwa Health Area.**

**Figure 2:**
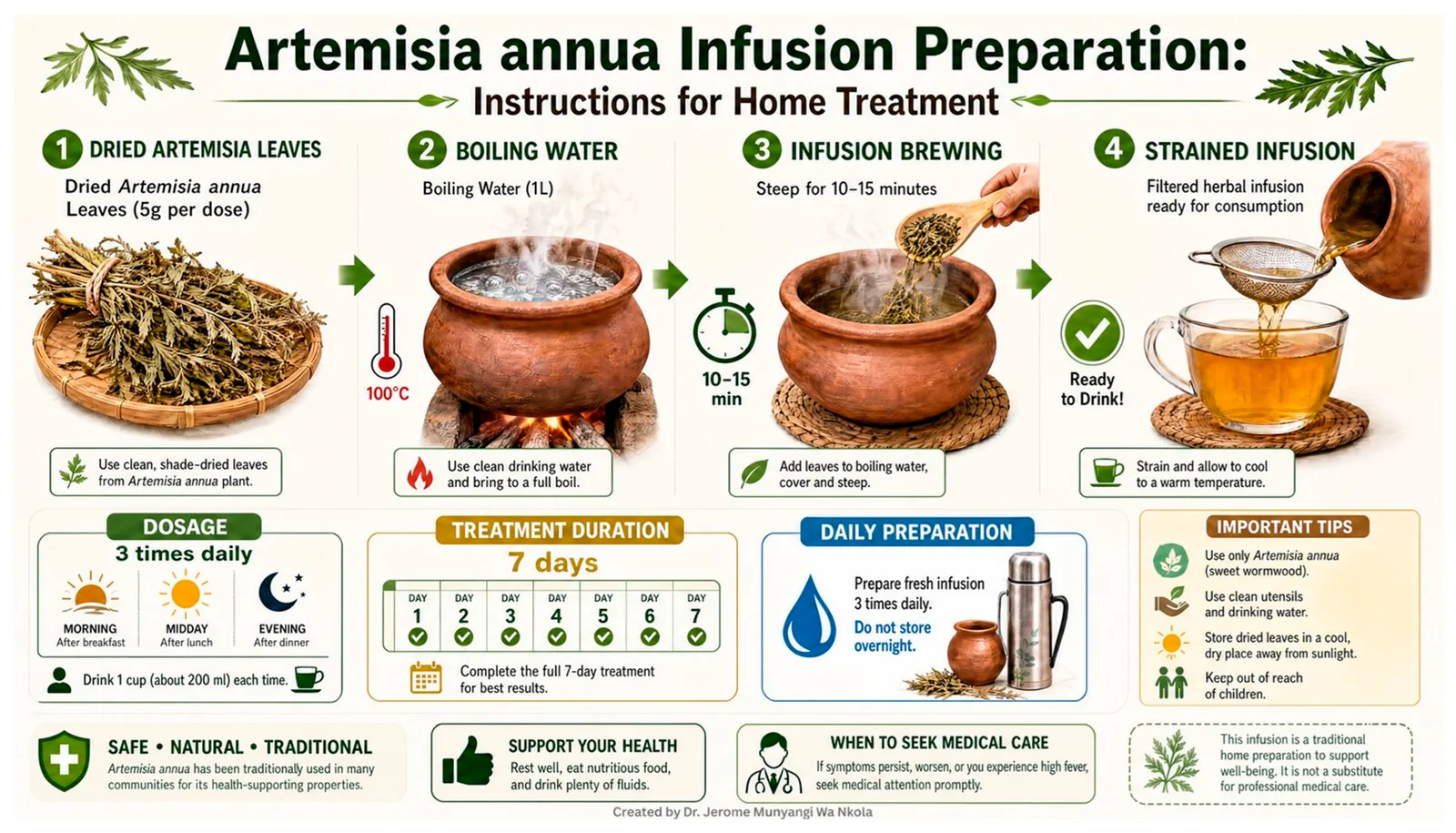
Standardized protocol for the domestic preparation and administration of *Artemisia annua* infusion.

The standardized visual aid previously labeled Figure 2 has been moved to Annex 1 to align with WHO-mandated cautions against unstandardized *Artemisia* monotherapy [36,37]. The substantive ethnographic content is preserved in narrative form there.

The infographic details the five-step process: harvesting, boiling, brewing, straining, and dosing. It emphasizes the 7-day treatment course and the thrice-daily administration schedule. While providing a structured guide, this visual also highlights the inherent challenges of maintaining precise dosage, particularly with “uncalibrated monotherapy” in rural household settings. Source: **Created by Dr. Jerome Munyangi Wa Nkola**.

As shown in Figure 2, the reliance on domestic preparation introduces significant variability in the concentration of active compounds. Unlike standardized ACTs, which provide a precise and stable dose of artemisinin derivatives [12], artisanal infusions of Artemisia annua are subject to fluctuations based on soil quality, harvest timing, and preparation methods 48. This lack of standardization frequently results in the delivery of sub-therapeutic doses, which, as documented in recent scoping reviews, creates the selective pressure necessary for the emergence and expansion of resistant Plasmodium strains [14]. This biochemical inconsistency remains a primary reason why international health consensus discourages the use of raw biomass for malaria management in endemic regions [48].

#### 3.2.3. Consumer Profile and Community Dynamics

**Vulnerable consumer strata**. Within the family ecosystem, *Artemisia* is conceptualized as a universal household-level therapeutic shield, with emphasis on pediatric and maternal utilization reflecting the perceived severity of malaria complications. Caregivers’ prioritization is influenced by the documented prevalence of non-compliant antimalarials in eastern DRC markets [7].

*“When young children take this remedy regularly, I sincerely believe that it can serve as a highly effective mechanism to help build widespread trust.”* (“*Quand les enfants prennent ça régulièrement, je crois que ça peut être un moyen très efficace…*”)

**BHP-04, Kinkungwa Health Area.**

*“I must specify the deep impact… It is a medication that directly influences the reduction of anemia rates, both in pregnant women and young children.”* (“*…c’est un médicament qui influence pour diminuer le taux d’anémie…*”)

**BHP-02, Kinkungwa Health Area.**

*“We often recall its collective administration to young children, and it was wonderful… The children would line up, each one holding a little cup.”* (“On se rappelle souvent de son administration collective aux enfants… Les enfants, chacun avec son gobelet”)

**CEU-03, Kakutya 2 Health Area.**

**Frequency of use and communal social acceptability**. The acceptance of this remedy has reconfigured local health landscapes, with diffusion described as “avenue by avenue.” Healthcare providers observed a concomitant decrease in outpatient attendance during periods of high botanical availability, suggesting that community-led modalities may temporarily alleviate pressures on fragile infrastructures.

*“This plant gained immense popular enthusiasm… We used to call out from one avenue to the next… for people to come and receive the product. The population was highly motivated.”* (*“Cette plante a gagné l’engouement populaire. On appelait une avenue par l’autre…”*)

**BHP-01, Bobela Health Area.**

*“We explicitly noted a frequency of usage so high that it directly impacted our formal hospital statistics… people no longer came to the hospital.”* (“*…nous notons une fréquence d’usage telle qu’elle impactait les statistiques hospitalières.*”)

**BHP-05, Bobela Health Area.**

*“Despite the complete termination of the institutional project, the public demand remains extremely high… the population is literally crying out for Artemisia.”* (*“Malgré l’arrêt du projet, la demande reste forte… les gens pleurent de l’artémisie”*)

**BHP-06, Bobela Health Area.**

### 3.3. Acceptability and Social Representations

The qualitative analysis also explored the community’s perceptions regarding the social acceptability of interventions and their preferences for specific vaccine formats. These detailed findings, structured according to the HBM-BeSD frameworks, are synthesized and presented in Table 6. 

#### 3.3.1. Social Acceptability and Therapeutic Rationality

The social acceptability of *Artemisia* is fundamentally rational and pragmatic rather than passive tradition, mapping to BeSD *thinking and feeling* [18]. The local population validates the remedy because it aligns with immediate, observable therapeutic benefits. Because a documented proportion of antimalarials in the region is substandard or falsified [6], the community perceives the botanical remedy as empirically more reliable than synthetic drugs of uncertain provenance [5].

*“This natural remedy gained immense popular enthusiasm because people witnessed the results directly on their sick relatives.”* (*“Ce remède naturel a gagné un engouement populaire immense parce que les gens ont vu les résultats directement sur leurs proches malades.”*)

**OIL-01, Kakutya 1 Health Area.**

*“The collective acceptance was built on visible facts. When people saw that others were recovering completely without incurring significant expenses, the whole community embraced it.”* (“*L’acceptation collective s’est construite sur des faits visibles. Quand les gens ont vu que les autres guérissaient complètement sans dépenser des fortunes, toute la communauté a adhéré.*”)

**CEU-02, Bobela Health Area.**

**Acceptance by necessity in the context of supply chain failure**. Under the BeSD *practical issues lens*, acceptability is amplified by macrostructural constraints [18]. In an enclaved territory, the community’s acceptance of *Artemisia* is a household-level response to documented supply chain failures [24].

*“Our acceptance is also a choice of survival. When the pharmacies in our health centers are completely empty, we naturally turn to what is available and effective in our gardens.”* (“*Notre acceptation est aussi un choix de survie. Quand les pharmacies de nos centres de santé sont complètement vides, nous nous tournons naturellement vers ce qui est disponible et efficace dans nos jardins.*”)

**CEU-04, Kinkungwa Health Area.**

*“It became a resource of solidarity and vital importance; the enclavement left us no other choice than to master the use of this plant to protect our households.”* (“*C’est devenu une ressource solidaire et vitale; l’enclavement ne nous laissait pas d’autre choix que de maîtriser l’usage de cette plante pour protéger nos ménages*”)

**OIL-03 Lubile Health Area.**

#### 3.3.2. Preferences Regarding the Medicine’s Galenic Form

This category explores the cognitive barriers and motivational drivers related to the physical presentation of the remedy, mapping the Health Belief Model dimensions of perceived barriers versus self-efficacy and the Behavioral and Social Drivers pillars of motivation and thinking and feeling [18].

**Rejection of the raw leaf biomass and artisanal constraints**. Both users and providers express strong aversion to the raw botanical format. The artisanal processing requiring precise boiling times and water volumes is a continuous source of posological anxiety. This operational complexity often leads to sub-therapeutic dosing, a recognized driver of antimalarial drug resistance [14,15], The logistical burden is exacerbated by documented supply chain bottlenecks in eastern DRC [24].

*“Those leaves there, we doubt them. The raw format is complicated because you can never be entirely sure if you boiled it too much or not enough.”* (“Les faits [feuilles], là, on doute. Le format brut est compliqué…”)

**CEU-05, Kakutya 2 Health Area.**

*“As healthcare technicians, we look down on the raw leaf format. It looks informal, and it makes the standardization of the active compound impossible in the field.” (“En tant que techniciens de la santé, nous voyons d’un mauvais œil le format des feuilles brutes.”*)

**BHP-03, Kalima General Hospital.**

**Consensus for pharmaceutical tablet forms**. Across all stakeholders, consensus exists for industrial galenic transformation [19]. The demand for tablets represents the ultimate trigger for full clinical self-efficacy. This preference reflects community perception of an unreliable formal pharmaceutical market, consistent with documented regional patterns of non-compliance [6,7]; a calibrated industrially produced *Artemisia tablet* is perceived as a guaranteed alternative that bypasses both artisanal error and non-compliant synthetic drugs [6].

*“My position is categorical regarding industrial processing. The moment they transform this plant into standard tablets, I will be the very first to consume it and give it to my children.”* (“Ma position est catégorique concernant la transformation industrielle…”)

**OIL-02, Kakutya 1 Health Area.**

*“If the state or pharmaceutical projects can process these plants into calibrated tablets, all professional hesitation will disappear because the precise dose will finally be guaranteed.”* (“Si l’État ou des projets pharmaceutiques peuvent transformer ces plantes en comprimés calibrés…”)

**BHP-01, Kinkungwa Health Area.**

*“We need this in a form that looks like real medicine. If it is in a tablet, the community will trust it even more than the ACTs we find in the market that sometimes do not work.”* (“Nous avons besoin de cela sous une forme qui ressemble à un vrai médicament…”)

**CEU-01, Bobela Health Area.**

#### 3.3.3. Social Dialog, Tensions, and Influential Leaders

**Religious impediments, structural doubts, and digital misinformation**. In the absence of institutional public communication, the use of raw leaves can reactivate traditional spiritual anxieties, with influential religious figures framing the remedy as a vehicle for occult practices. Contemporary religious influence in the region often uses digital platforms to propagate sophisticated health narratives and misinformation, a dynamic observed in fragile healthcare systems with low regulatory oversight [49]. Integrating behavioral science into intervention design is therefore essential to address the social processes that drive or inhibit adoption [18].

*“In my specific religion, some leaders create deep doubts. They tell the congregation that we might be transforming these raw leaves using sorcery or witchcraft.”* (“Dans ma religion spécifique, certains dirigeants créent des doutes profonds…”)

**CEU-05, Kakutya 2 Health Area.**

*“Without official sensitization from the authorities, people will continue to listen to certain pastors who claim there is magic behind this natural remedy.”* (“Sans une sensibilisation officielle des autorités, les gens continueront d’écouter certains…”)

**BHP-02, Bobela Health Area.**

**Biomedical validation channels versus household-level agency**. Structural authority figures can act as powerful cues to action [19]; the documented regional prevalence of non-compliant antimalarials and local perception of unreliable supplies have eroded community trust in formal pharmacies [6,8]. When formal medicines are perceived as unreliable or physically absent (the *tyranny of empty shelves* [5]), the collaborative endorsement of local physicians and trained pastors becomes the primary driver for community-wide trust [42].

*“I suggest the extreme importance of religious leaders… when we formally train the pastors, it automatically influences and reassures their followers.”* (“Je suggère l’importance extrême des leaders religieux…”)

**OIL-01, Lubile Health Area.**

*“We need to break the conflict between the clinic and the community. When local physicians and socio-religious figures validate the remedy together, the population follows without fear.”* (“Nous devons briser le conflit entre la clinique et la communauté…”)

**BHP-01, Bobela Health Area.**

### 3.4. Structural Impacts and Social Equity

This final axis evaluates the systemic repercussions of integrating *Artemisia annua into* the local healthcare landscape, focusing on hospital efficiency, economic justice, and the demand for institutional sovereignty. The data reveal that botanical therapy acts as a buffer against the two-fold failure of the formal health system: the physical absence of medicine (the **“tyranny of empty shelves”** [5]) and the prevalence of fraudulent products in the market [6].

Table 7 provides the analytical structure for evaluating structural resilience and financial equity, mapping how community-led production can mitigate the inequities caused by a compromised pharmaceutical market.

#### 3.4.1. Structural Impacts on the Healthcare Ecosystem and Social Equity

**Reduction of hospital overcrowding**. Informants established a perceived link between community-wide use of the infusion and a decrease in severe malaria cases reaching formal facilities, allowing fragile health centers to focus resources on other critical pathologies.

*“We felt that it has at least decreased the frequency of malaria patients in our wards.”* (“On a senti que ça a au moins diminué les taux de fréquence des malades qui ont du paludisme…”)

**BHP-01, Bobela Health Area.**

*“Thanks to this plant, the populations no longer went up to the hospital to be treated for this disease, which significantly lightened our clinical burden.”* (“*Grâce à la plante les gens ne montaient plus encore à l’hôpital pour être traités avec cette maladie, ce qui a largement allégé notre charge clinique.*”)

**CEU-02, Bobela Health Area.**

**Economic resilience**. Under the BeSD *practical issues lens*, the primary structural value of *Artemisia* serves as a lever for socioeconomic justice. In a hyper-vulnerable context shaped by conflict and supply chain failures, conventional therapies are often economically prohibitive [4,24]. By offering an autonomous, zero-cost remedy cultivated within the domestic perimeter, the plant successfully lifts the financial barriers that historically excluded the poorest households from formal care. This self-production acts as a safeguard against the documented regional patterns of poor-quality and non-compliant pharmaceutical products [8].

- *“Artemisia stands as a vital resource for our most precarious populations. Its absolute freeness and geographic proximity transform it into an essential therapeutic pillar, guaranteeing treatment with zero financial barriers for impoverished families.” (“Sa gratuité absolue et sa proximité géographique en font un pilier thérapeutique essentiel garantissant le traitement avec zéro barrière financière pour les familles pauvres.”)* CEU**-04, Kinkungwa Health Area**.
- *“I view Artemisia as a powerful lever for social equity. By being accessible completely free of charge, it entirely lifts the financial burden of healthcare, offering the poorest communities an autonomous therapeutic solution in the face of medicines.” (“Je vois l’Artemisia comme un puissant levier d’équité sociale. En étant accessible complètement gratuitement, elle lève entièrement le fardeau financier des soins…”)* BHP-03, Lubile Health Area.

#### 3.4.2. Perspectives for National Health-Policy Integration

This category highlights a structured political awareness among the key actors of Kalima, reflecting high motivation for institutional change.

**Demands for official validation and integration**. Informants challenged the central Congolese state and the Programme National de Lutte contre le Paludisme to resolve the historical paradox surrounding the plant. There is a unified demand for formal institutional validation, positioning state recognition as the ultimate cue to action required to legally integrate *Artemisia into* rural health centers and eliminate professional clinical hesitancy. This demand aims to secure a local, reliable supply chain that is resilient to the recurrent geopolitical shocks affecting the province [5].

*“I directly challenge the authorities regarding the contradiction between our proven field efficacy and their complete lack of institutional support. Why do the health policymakers of our country refuse to officially integrate this product into our community health centers?” (“J’interpelle directement les autorités sur la contradiction entre notre efficacité prouvée et leur absence totale de soutien institutionnel.”)*

**BHP-01, Kakutya 1 Health Area.**

*“We need a clear, formal decision from the Ministry of Health. If the PNLP officially includes Artemisia in its protocols, local healthcare workers will finally be able to prescribe it without fear or professional reservation.” (“Nous avons besoin d’une décision formelle claire du Ministère de la Santé …les professionnels de la santé locaux pourront enfin la prescrire sans crainte ni réserve professionnelle.”)*

**BHP-03, Kinkungwa Health Area.**

**Local production strategies and community sovereignty over supply chains**

**Local production strategies**. To prevent recurrent stockouts that frequently follow the departure of international aid projects in the DRC, participants advocate for agricultural and technical self-reliance.

*“We must learn the lessons from the post-project stockouts. The community must be empowered to cultivate, harvest, and preserve the plant autonomously so that we are never again dependent on external actors.”* (“Nous devons tirer les leçons des ruptures de stock post-projet. La communauté doit être autonome pour cultiver, récolter et conserver la plante […]”)

**OIL-02, Lubile Health Area.**

*“True therapeutic sovereignty for Kalima lies in our capacity to organize local production. By training agricultural and health relays together, we build a permanent community shield against malaria.” (“La véritable souveraineté thérapeutique pour Kalima réside dans notre capacité à organiser la production locale. En formant ensemble les relais agricoles et sanitaires, nous construisons un bouclier communautaire permanent contre le paludisme.”)*

**BHP-02, Bobela Health Area.**

**Analytical synthesis**. Community motivation is no longer solely dependent on external project stimuli but also on the perceived necessity of self-efficacy in a fragile-state environment [24]. By framing state validation as a cue to action, participants articulate a path toward bridging the gap between policy and practice. The transition from aid dependence to community-managed production is positioned as a practical response to recurring supply chain bottlenecks documented in the Maniema region [4] (Table 8).

## 4. Discussion

This Discussion operates in strict dialogical mapping with Section 3 *Results* (Table 3, Table 4, Table 5, Table 6, Table 7 and Table 8 and verbatim quotations in Section 3.1.1 through Section 3.4.2). Section 4.4 concentrates the dialogical integration of the six inductive themes consolidated in Section 2.5.2: (i) religious mediation of pharmaceutical choices; (ii) calendar alignment with seasonal malaria peaks; (iii) the *“Hilary pattern”* of recalled collective under-5 administration; (iv) Marie Stopes/intergenerational memory of pre-WHO policies; (v) intra-household conflict over phytotherapy; (vi) professional category variation in provider tolerance, with Section 4.5 and Section 4.7 re-anchoring the same themes to public health implications and reflexivity. The integrated HBM-BeSD framework mapping installed in Section 3 is preserved here uniformly across the four WHO BeSD domains [*thinking and feeling/social processes/motivation/practical issues*] [18]. Numerical figures quoted from [22,43] and are preserved verbatim and are framed as contextualizer evidence drawn from the same source population, not as a methodological integration into the present qualitative submission. The empirical-vs-operational distinction introduced in Section 5.1 and Section 5.2 is preserved here in Section 4.5 (empirical consequences) and Section 4.6 (operational recommendations), with internal cross-references to Section 5.2 recommendations (i)–(vi) marked explicitly.

### 4.1. Synthesis: Behavioral Reality vs. Clinical Boundaries

This qualitative investigation explored the perceptions and social representations of *Artemisia annua* herbal tea within the Kalima Health Zone of Maniema Province, anchoring the analysis on the integrated HBM-BeSD framework [18,19]. The mapping installed in Section 3—across Table 3, Table 4, Table 5 and Table 6—establishes that adoption is a household-level pragmatic response to severe structural constraints, not a behavioral manifestation of traditional belief. The verbatim closing of Section 3.4.2 captured by an opinion leader (OIL-02, Kakutya 1: “*the very first to consume it and to give it to my children*”), a biomedical provider (BHP-01, Kinkungwa: “*all professional hesitation will disappear because the precise dose will finally be guaranteed*”), and a community end-user (CEU-01, Bobela: “*a form that resembles a real medicine… so that the community will trust it even more than the ACTs we find at the market, which sometimes do not work*”) frames Section 4.1’s opening synthesis: across three functional strata (CEU/BHP/OIL), the transversal consensus demands industrial galenic transformation, not endorsement of raw biomass ingestion.

It is essential to emphasize that this study is strictly socio-behavioral. It analyzes community drivers and perceptions and does not constitute a clinical trial, nor does it provide scientific validation of the clinical efficacy, safety, posological adequacy, or resistance-attenuation potential of *A. annua* herbal tea [12,13,36,37]. In accordance with international guidelines, artemisinin-based combination therapies remain the only validated first-line molecules for uncomplicated *Plasmodium falciparum* (*P. falciparum*) malaria. The reliability of the findings is supported by the operational achievement of thematic saturation through the three procedures documented in Section 2.5.3, following qualitative saturation recommendations [38,40,41,44]; the N = 30 cohort (11 CEU/11 BHP/8 OIL)—equally distributed across the five *aires de santé* (Bobela, Kakutya 1, Kakutya 2, Kinkungwa, Lubile) at the 20.0% per-site target constituted the analytic substrate whose inter-stratum triangulation produced the four operational axes reported in Section 3.

### 4.2. Risks of Resistance and the Status of Uncalibrated Monotherapy

A significant point of tension concerns the gap between the perceived rapid recovery documented in Section 3.1.2 and Section 3.3.1 (BHP-01, Bobela: “*we felt that it has at least decreased the frequency of patients suffering from malaria*”; OIL-03, Kakutya 1: “*it achieves a cure in 3 days, whereas the modern clinic protocol requires 7 days*”) and the underlying biological risks of resistance. The WHO classifies *Artemisia* infusions as unstandardized monotherapy whose use is currently discouraged to prevent the selection and spread of resistant parasites [36,37]. Variable concentrations of active ingredients in artisanal preparations create selection pressure for resistance [15]. In the Kalima cohort specifically, the documentation of CEU-06 (Kakutya 2: “*nothing more than boiling it and then I drink it*”) and BHP-07 (Bobela: “*we always dried it strictly in the shade*”) in Section 3.2.2 confirms empirically the household-level posological heterogeneity that pre-clinical literature identifies as a primary resistance driver [46,47].

Sub-therapeutic dosing—whether through non-quality-assured synthetic drugs or uncalibrated botanicals—is an established source of antimalarial drug resistance that operates in the same regional epidemiological landscape as the recent surveillance in neighboring East Africa confirming the expansion of *P. falciparum* strains with artemisinin partial resistance [14]. The expanding frontier of documented resistance markers, including the pre-clinical WHO-validated pfk13 propeller mutations whose first molecular evidence was recently confirmed in the Kalima Health Zone itself, underscores that population-level exposure to sub-therapeutic artemisinin doses—through any vector—must be treated as a coupled epidemiological risk rather than as a peripheral concern. The verbatim posological anxiety registered in Section 3.2.2 (BHP-03, Kinkungwa: “*we, the formal health technicians, are afraid… we hesitate*”) is therefore a legitimate perception, grounded in the underlying pharmacological asymmetry between standardized ACT formulations and artisanal *Artemisia* decoctions. The bivalent position taken by Section 5.1—that the population has converted a documented systemic failure into a household-level therapeutic routine, and now demands that this routine be both standardized and integrated into the formal pharmaceutical system—follows directly from the empirical triangulation reported in Section 3 and is preserved here without modification.

### 4.3. Geopolitical Context, Supply Chain Failures, and Falsified Medicines: Separating Documented Evidence from Community Perceptions

The resort to botanical alternatives documented in Section 3.2.1, Section 3.3.1 and Section 3.4.1 stems from a dual systemic failure of the pharmaceutical supply chain in Maniema Province [4], and from a community-perceived unreliability of the pharmaceutical market consistent with documented regional patterns of ACT non-compliance. This finding provides context for Section 3.3.2 (BHP-03, Kalima General Hospital: “*we look down on the raw leaf format*”) and Section 3.4.2 (OIL-02, Lubile: *“the community must be empowered to cultivate, harvest, and preserve the plant autonomously”*) the verbatim rejection of raw biomass being motivated not by botanical skepticism but by a perception of inferior galenic reliability relative to the formal pharmaceutical market, while the demand for community-managed local production constitutes a structural response to the recurrent stockouts documented in Section 3.2.1 (BHP-06, Bobela: “*the population is literally crying out for Artemisia*”).

This section employs a four-category explicit triangulation, separating documented regional evidence from community perceptions of pharmaceutical quality at the study site. The present Section 4.3 applies a four-category triangulation discipline that rigorously distinguishes **documented regional pharmaceutical-quality evidence** [6,9]; **structural patterns of supply chain disruption documented at the provincial level** [3,4]; **community perceptions of pharmaceutical quality at the Kalima study site**, captured by Section 3.3.1 (CEU-04, Kinkungwa: “*the pharmacies in our health centers are completely empty*”) and Section 3.3.2 (CEU-01, Bobela: *“ACTs we find in the market that sometimes do not work”*) and triangulated across CEU/BHP/OIL; and **the empirical reality of pharmaceutical prevalence at Kalima**, which the present study does **not** establish empirically. This categorical separation constitutes one of the operative safeguards against the holistic bias that the qualitative methodological literature explicitly identifies as a risk in studies of perceived health-system failure [25,26].

**Category 1—Documented regional evidence**. Studies in Bukavu (Mahano et al., 2021, 2026) document that a significant proportion of antimalarials, including quinine and artemether-lumefantrine, fail to meet essential quality standards [6,7]. A Kinshasa-based study has highlighted that the lack of robust regulatory oversight in the DRC allows for the widespread sale of non-compliant pediatric formulations supplied through informal private wholesalers [8]. The SADC region has formally identified the prevention, detection, and response to substandard and falsified medical products as a regional priority [10]. Nationally, a study covering eight cities of the DRC documented non-compliance in artemether-lumefantrine formulations [9]. HPLC and UV-Vis analyses on antibiotics sold in the DRC confirm that the country faces a generalized problem of substandard medications affecting multiple therapeutic classes [11].

**Category 2—Documented structural provincial patterns**. Recurrent stockouts of essential biomedical countermeasures in Maniema are documented across the eastern DRC literature [3,4,5]. The verbatim CEU-08 in Section 3.2.1., *“I express deep regret for never having been able to test the product… I have simply never had the opportunity to physically touch this medication”*, illustrates the convergence of post-project stockouts and community demand that Section 3 documents at the household level.

**Category 3—Community perceptions at Kalima**. Participants reported observations of empty pharmacies and unreliable ACTs (Section 3.3.1 and Section 3.3.2). These perceptions were triangulated across biomedical providers, end-users, and opinion leaders, strengthening their *internal consistency*, but—by the explicit methodological posture of the present submission—they do not establish their *external validity* as prevalence estimates.

**Category 4—Unknown empirical prevalence at the study site**. The present study does *not* measure parasitological outcomes, does *not* test the chemical quality of antimalarials sold at Kalima, and does *not* establish the prevalence of substandard ACTs at the study site. All statements on pharmaceutical quality in Kalima are framed as community perceptions extrapolated from documented regional patterns. The exact distribution of textual evidence across the four categories is detailed verbatim by the Bukavu quality citations [6,7], the Kinshasa pediatric citation [8], the 8-city national survey citation [9], the HPLC/UV-Vis DRC citation [11], the SADC priority citation [10], and the adjacent East-African resistance surveillance citations [14].

**Author interpretation**. The convergence of documented regional evidence (Categories 1 and 2) and triangulated community perception is consistent with but does not constitute proof of significant pharmaceutical quality failures in the Kalima Health Zone (Category 4 unresolved). Future studies should conduct HPLC or Minilab-based pharmaceutical surveillance at the study site to test this hypothesis empirically, as foreshadowed in Section 5.4, future research priority #2.

**Explicit non-validation disclaimer**. The present study is strictly socio-behavioral. It does *not* validate the clinical efficacy, safety, dosing adequacy, or resistance-attenuation potential of *A. annua* herbal tea. The disincentive framework anchored on [12,13,36,37] is preserved verbatim: WHO discourages unstandardized monotherapy [12,13]; WHO-cited Ugandan medicinal plant evidence establishes that documented efficacy in standardized extracts cannot be presumed to transfer to artisanal teas [37]; and *Artemisia*-independent inhibitory activity against hypnozoites [36] emphasizes that any standardized formulation would require rigorous pharmacological characterization.

**Figure 3:**
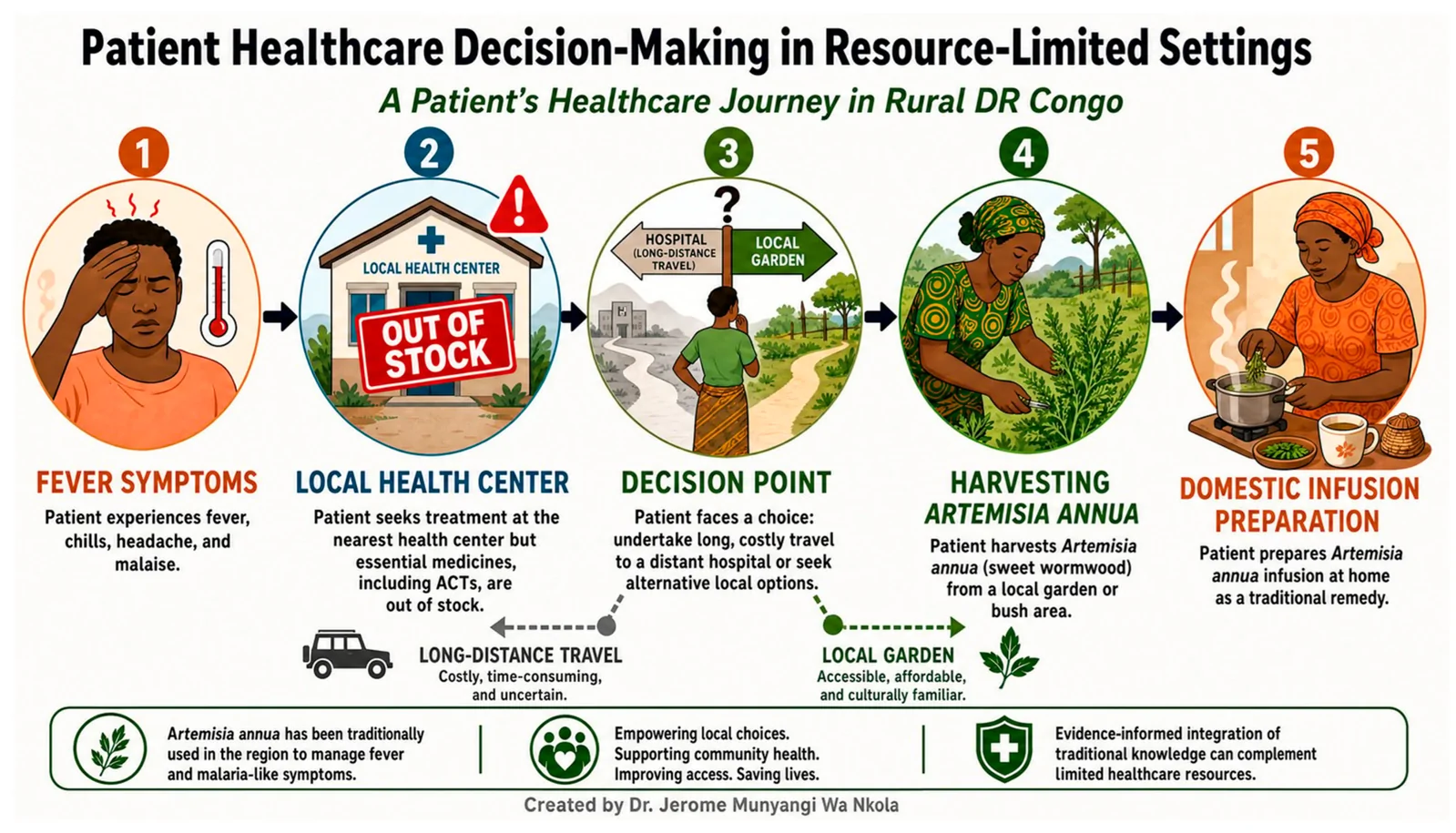
Patient healthcare decision-making and the path to therapeutic resilience in Kalima District.

**Figure 3—Patient healthcare decision-making and the path to therapeutic resilience in Kalima District**.

The infographic depicts the five-stage transition from symptom onset to domestic herbal tea preparation. Stages 2 and 3 highlight the critical failures in the formal supply chain (stockout events) and the erosion of trust due to the prevalence of substandard drugs that necessitate a pivot toward local resources like *Artemisia annua*. This trajectory illustrates how physical enclavement and supply chain bottlenecks transform a botanical alternative into a primary strategy for structural survival. *Source: Created by the author for this study*.

The infographic depicts the five-stage transition from symptom onset to domestic herbal tea preparation. Stages 2 and 3 highlight the critical failures in the formal supply chain (“Out of Stock” events) and the erosion of trust due to the prevalence of substandard drugs that necessitate a pivot toward local resources like *Artemisia annua. This* trajectory illustrates how physical enclavement and supply chain bottlenecks transform a botanical alternative into a primary “Structural Survival” strategy [5,11,37].

*Source: Created by Dr. Jerome Munyangi Wa Nkola*.

**Stage** **2: The Infrastructure Failure**

The “Out of Stock” sign depicted in Figure 2 represents a widespread reality in Maniema, where facilities frequently lack essential antimalarials [5,6]. However, this “Physical Vacuum” is compounded by a “Quality Vacuum” the high prevalence of substandard and falsified medicines in the DRC [9,10]. This dual failure forces patients to seek alternatives, as even available synthetic drugs are often perceived as ineffective or fraudulent [11].

**Stage 3: The Convergence of Social Drivers**

This decision point is where **WHO-BeSD Social Processes** [36] and personal beliefs converge. Faced with the impossibility of accessing reliable formal treatment, the “local garden” becomes the only rational and accessible option for an isolated population [11]. This choice is driven by “Pragmatic Rationality,” where the community-validated efficacy of the plant outweighs the risks of an unreliable formal market.

**Stage 5: Domestic Preparation and Public Health Risks**

Step 5 highlights the domestic preparation process, which, while empowering for the household, raises crucial public health questions. The use of uncalibrated household utensils introduces significant variability in dosing. This raises concerns regarding **sub-therapeutic concentrations** and the potential for selecting resistant parasite strains, a risk particularly relevant given the emergence of resistance markers in neighboring East African regions.

### 4.4. Social Dynamics and Belief Models

The integration of the HBM and BeSD frameworks provides a critical interpretive lens through which to understand why community behavior diverges from national protocols despite high awareness of malaria severity [50], an observation analyzed through the HBM and BeSD frameworks [18]. Section 4.4 details the dialogical integration of the **six inductive themes** (i)–(vi) consolidated in Section 2.5.2 and re-anchored in Table 3 (last row) and Table 8 of Section 3 themes that emerged outside the deductive HBM-BeSD grid and that Section 4.5 and Section 4.7 subsequently re-anchor to public health implications and reflexivity. Each of the six inductive themes is mapped here to specific Section 3.1.1 and Section 3.1.2 verbatim anchors, with Section 3 BeSD domain notation preserved.

**Theme (i)—Religious mediation of pharmaceutical choices (BeSD social *processes*)**. This theme dialogically re-anchors Section 3.3.3 (CEU-05, Kakutya 2: *“some leaders create deep doubts. They tell the congregation that we might be transforming these raw leaves using sorcery or witchcraft”*) and Section 3.3.3 (OIL-01, Lubile: *“I suggest the extreme importance of religious leaders… when we formally train the pastors, it automatically influences and reassures their followers”*). The bidirectional valence of religious mediation simultaneously amplifying and opposing—is independently confirmed by the divergent testimony OIL-06 (Lubile, Section Divergent Cases Identified Through Negative Case Analysis: *“the leadership forbids the use of this remedy because it is associated with occult rituals”*). This bidirectional posture is a structural feature of the *social processes domain* of BeSD [18]; it cannot be reduced to a unidirectional cue-to-action channel. The complexity and shifting nature of community perceptions of public health interventions, including malaria vaccination in Africa, has been documented in recent scoping reviews [49]. The implicit asymmetric handling of religious mediation in many public health interventions constitutes a documented operational blind spot in fragile healthcare systems, which Section 5.2 recommendation (iii) addresses symmetrically.

**Theme (ii)—Calendar alignment with seasonal malaria peaks (BeSD practical issues)**. This theme dialogically re-anchors Section 3.2.1 (BHP-05, Bobela: *“we had instructed people to drink it every single morning… we noticed a significant decrease in the number of individuals showing clinical signs of malaria*”) and Section 3.2.1 (BHP-06, Bobela: *“its role lies in malaria prevention, while for patients, its primary role remains the clinical treatment”*). The verbatim testimony of BHP-05 documents the calendar-aligned preventive administration of the infusion, anchoring this inductive theme to the *practical issues* domain of BeSD [18]—a structured household-level adaptation to documented seasonality that operates independently of formal public health communication. The corresponding relapse-causing hypnozoite activity documented in [36] underscores that the *A. annua* infusions cannot be presumed to substitute for formal prophylaxis, even where calendar alignment is empirically observed.

**Theme (iii)—The “*Hilary pattern*” *of* recalled collective under-five administration (BeSD *thinking and feeling*)**. This theme dialogically re-anchors Section 3.2.3 (CEU-03, Kakutya 2: *“we often recall its collective administration to young children… The children would line up, each one holding their own little cup”*) and Section 3.2.3 (BHP-04, Kinkungwa: *“when young children take this remedy regularly, I sincerely believe that it can serve as a highly effective mechanism to help build widespread trust”*). This recalled collective administration pattern operationally designed for vulnerable pediatric and maternal demographics (HBM severity/BeSD *thinking and feeling*) [18,19] is a first-rank inductive code in Table 8 and is preserved here as a structural pattern that any future galenic transformation (Section 4.6 recommendation (ii)) would have to address in terms of posological adequacy for pediatric populations, which the WHO-cited Ugandan medicinal plant evidence establishes cannot be presumed absent dedicated pharmacokinetic characterization [37].

**Theme (iv)—Marie Stopes/intergenerational memory of pre-WHO policies (BeSD social *processes*)**. This theme is preserved here as a constitutive memory trace anchoring household-level therapeutic agency within a transgenerational frame. The verbatim BHP-03 (Lubile: *“I view Artemisia as a powerful lever for social equity”*) *and* CEU-02 (Bobela: *“the collective acceptance was built on visible facts”*), from Section 3.4.1, articulate the post-WHO intergenerational memory through which the *A. annua* infusion has been integrated into household therapeutic sovereignty. This intergenerational channel is consistent with documented patterns of decentralized knowledge co-production in fragile states [51]. Operationally, it informs—but does not transform—recommendation (ii) of Section 4.6 by establishing the depth of household demand that any standardized galenic product must satisfy in order to maintain downstream adoption.

**Theme (v)—Intra-household conflict over phytotherapy (BeSD *thinking and feeling/motivation*)**. This theme dialogically re-anchors Section 3.2.1-b divergent testimony CEU-08 (Lubile: “*I prefer to use ACTs at the clinic. I don’t trust leaves that come from the bush*”), the structural-reasons-for-non-usage Section 3.2.1 (CEU-08: *“I have simply never had the opportunity to physically touch this medication”*), and Section 3.3.3 (BHP-02, Bobela: “*Without official sensitization from the authorities, people will continue to listen to certain pastors who claim there is magic behind this natural remedy*”). This intra-household conflict pattern intersects with the *Hilary pattern* (theme iii) at the level of contested household authority over pediatric decision-making. Such intra-household conflict represents a structural feature of decentralized therapeutic ecosystems in fragile states, consistent with the medical-pluralism frame (Kleinman) and observations on health service provision at the margins [24].

**Theme (vi)—Professional category variation in provider tolerance (BeSD thinking *and feeling/motivation*)**. This theme dialogically re-anchors Section 3.2.2 (BHP-02, Kinkungwa: *“I strongly prefer the transformed pharmaceutical presentation for the absolute safety of the dosage”*) and Section 3.2.2 (BHP-03, Kinkungwa: *“we, the formal health technicians, are afraid… we hesitate”*), and is independently confirmed by the divergent testimony BHP-04 (Kinkungwa, Section Divergent Cases Identified Through Negative Case Analysis: *“as a professional, I would never recommend a tea without knowing the dosage. It is risky for our patients”*). The professional category decomposition in Table 2 of Section 2.4.2—one physician, five Infirmiers Titulaires, and five midwives within the BHP stratum—provides the structural substrate for this variation: respondents differ in their degree of autonomy to prescribe, in their exposure to pharmaceutical posological training, and in their relative weight of clinical hesitancy versus pragmatic demand for tablet-form alternatives. The verbatim CEU-09 (Lubile, Section 3.1.1: “*Honestly, I haven’t seen it yet… I have only heard about it, yes*”) and BHP-10 (Kalima, Section Divergent Cases Identified Through Negative Case Analysis: *“I have never personally used it, even though I know people who do”*) corroborate that this variation extends across the lay/provider boundary as well, capturing the full span of professional category variation in provider tolerance.

**Synthetic dialog with Section 3 cross-cutting Table 8**. Each of the six inductive themes above corresponds to one or several row entries of Table 8 in Section 3—geographic variation in familiarity (Section 3.1/*thinking and feeling), Hilary pattern* recall (Section 3.2/*practical issues*), divergent testimonies (Section Divergent Cases Identified Through Negative Case Analysis/*social processes*), *tablet preference* and *pure* perception (Section 3.3/*motivation*), and *local garden* preference and collective memory of post-project stockouts (Section 3.4/*practical issues*)—under the same HBM-BeSD construct notation. The mapping is preserved verbatim across Section 3 and Section 4 to allow auditability of the cross-section link.

### 4.5. Public Health Implications

The empirical results documented in Section 3 add to a growing alarm signal regarding the vulnerability of the healthcare system in Kalima. The present Section 4.5 preserves the **empirical-vs-operational distinction** introduced in Section 5.1 and Section 5.2: this section documents empirical consequences, while Section 4.6 articulates the corresponding operational recommendations. The empirical consequences are organized in three sub-points.

**Sub-point 1—Documented physical and quality dual failure**. While the physical gap of drug stockouts is a primary driver [4,5], the documented regional quality concern the prevalence of substandard and falsified medications across the SADC region and within the DRC [7,10], acts as a distinct push factor toward non-validated botanical remedies. For the PNLP, the challenge goes beyond simple logistics; it requires restoring the quality integrity of the antimalarial market to prevent the further marginalization of vulnerable populations [8]. The verbatim Section 3.4.1 CEU-04 (*“treatment with zero financial barriers for impoverished families”*) and Section 3.4.1 BHP-03 (*“entirely lifts the financial burden of healthcare”*) demonstrate that the household-level response is materially rooted in this dual failure and not in botanico-romantic endorsement a critical distinction for any policy actor attempting to interpret the present findings.

**Sub-point 2—Religious mediation as a bidirectional lever (re-anchoring of inductive theme (i) to public health implications)**. The SD-SADC literature identifies behavioral change communication modulated through religious and traditional leaders as a structural lever for therapeutic adoption [52]. In the present study, the verbatim symmetrical valence documented in Section 3.3.3 OIL-01 amplifying, CEU-05/OIL-06 opposing establishes that any public health intervention that does not engage religious leaders symmetrically will produce unintended effects in either direction. Future interventions must embed this bidirectional structure into their communication strategy from conception.

**Sub-point 3—Calendar alignment and pediatric vulnerability (re-anchoring of inductive themes (ii) and (iii) to public health implications)**. The collectively recalled under-five administration pattern (*Hilary pattern*, theme iii) and the calendar-aligned preventive administration pattern (theme ii) jointly establish a household-level preventive practice in pediatric populations that Section 3.2.3 documents as having once been clinically observed across the community. Any future galenic formulation developed under Section 4.6 recommendation (ii) must therefore undergo dedicated pediatric pharmacokinetic characterization before any public health deployment in the *Hilary pattern* configuration, in continuity with the cautions issued in [12,13,36,37] and with the documented regional concern of sub-therapeutic dosing observed in adjacent East African settings [14].

**Contextualizer evidence framing [22,43]**. As contextualizer evidence drawn from the same Kalima Health Zone source population, the present qualitative discussion is informed by two independently submitted cross-sectional surveys issued from the same research program. The provider survey [22] established that providers have recommended the herbal tea, and identified barriers to broader professional endorsement such as lack of galenic standardization and perceived ineffectiveness. The cross-sectional conclusion of the provider survey regarding clinical acceptability and efficacy [22] is preserved as the contextual numeric framework within which the present qualitative BHP cohort (n = 11) is interpreted.

The household survey (n = 619) demonstrates that infusion reliance functions as a rational socioeconomic coping mechanism, reaching 64.7% in the lowest wealth tertile and 77.8% in households of 16 members or more, with 88.2% of users reporting a high perceived community tolerance observations that frame the very concept of localized resilience advanced in the verbatim conclusion of [43]. These figures including n = 619, 64.7%, 77.8% and 88.2% are also preserved verbatim. The verbatim conclusion of the household survey that effective malaria control in fragile settings requires bridging the access gap for validated therapies while integrating community practices into standardized genomic and clinical surveillance systems articulates a programmatic recommendation directly compatible with the conclusions of the present manuscript, which calls for galenic standardization aligned with PNLP recommendations and the inscription of ethnographic research within an integrated genomic–clinical–behavioral surveillance architecture (Section 5.3).

**Crucial non-integration disclaimer**. These two numerical captures, drawn from independent submissions, do not enter the present manuscript as a methodological triangulation layer; they enter only as background contextualization that, taken together with the qualitative narrative depth of the three functional strata explored in this manuscript, substantively informs but does not methodologically merge with the public health implications advanced below. The present manuscript maintains its independent qualitative identity: any numerical figure quoted from [22] or [43] is preserved verbatim but remains strictly attributable to its source cross-sectional survey in which it was originally produced.

### 4.6. Implications and Recommendations

The present Section 4.6 articulates the **six operational recommendations** that map **exactly** to Section 5.2 (recommendations (i)–(vi)) of Section 5 Conclusions. The empirical-vs-operational distinction preserved in Section 4.5 and Section 4.6 is anchored on the architecture of Section 5.1 (main empirical conclusion) and Section 5.2 (operational recommendations): Section 4.5 documents the empirical consequences that the qualitative evidence establishes, while Section 4.6 translates those consequences into actionable policy levers. The internal cross-references to Section 5.2 are preserved verbatim.

**Recommendation (i)—Securing first-line pharmaceutical supply chains (cross-reference Section 5.2 (i))**. National policymakers and the Programme National de Lutte contre le Paludisme should prioritize logistical funding and secure overland transport routes in eastern DRC. Stabilizing consistent ACT availability at peripheral clinics is essential to prevent the documented pattern of empty shelves in conflict-affected zones [3,5]. This recommendation directly addresses the empirical pattern documented by the structural-reasons-for-non-usage Section 3.2.1 and re-anchored in Section 3.4.1 (CEU-04, Kinkungwa: *“treatment with zero financial barriers”*).

**Recommendation (ii)—Galenic and posological standardization research (cross-reference Section 5.2 (ii))**. State-led institutions should invest in evaluating local botanical resources using a reverse-pharmacology approach, focusing on the potential industrial transformation of raw biomass into standardized tablets [53]. This is necessary to eliminate posological anxiety (Section 3.2.2 BHP-03: *“the raw leaf format… it makes the standardization of the active compound impossible on the ground”*) and the documented risks of sub-therapeutic dosing [15,37]. The transversal demand for industrial galenic transformation documented in Section 3.3.2 (OIL-02, BHP-01, CEU-01) constitutes the qualitative evidentiary substrate of this recommendation.

**Recommendation (iii)—Symmetrical religious community engagement (cross-reference Section 5.2 (iii); re-anchoring of inductive theme (i))**. Public health authorities must actively involve trusted local opinion leaders, specifically training socio-religious figures and traditional healers as information champions [52]. These leaders should be equipped to dismantle spiritual rumors and disseminate evidence-based health choices. The symmetrical engagement architecture is mandated by the bidirectional religious valence documented in Section 3.3.3 amplification by OIL-01, opposition by CEU-05 and OIL-06.

**Recommendation (iv)—Targeted biomedical provider training (cross-reference Section 5.2 (iv))**. Formal capacity-building is required for healthcare workers regarding therapeutic pluralism. Guidelines must equip providers to address community phytotherapeutic practices while firmly upholding the mandatory priority of immediate clinical testing and the administration of validated ACTs [3]. The cross-sectional evidence from [22]—22.6% of providers citing lack of galenic standardization and 18.4% citing the risk of perceived ineffectiveness as principal barriers to broader professional endorsement—is preserved here as contextualizer evidence framing the need for this training.

**Recommendation (v)—Incorporation of negative cases into communication strategy (cross-reference Section 5.2 (v))**. The four divergent testimonies documented in Section Divergent Cases Identified Through Negative Case Analysis (CEU-08, BHP-04, OIL-06, BHP-10) are not residues to be discarded but constraints to be incorporated into policy design. CEU-08 establishes that ACT-first messaging remains strategically viable for a substantial minority. BHP-04 confirms that posological calibration is the primary structural constraint on professional endorsement. OIL-06 establishes that religious-dialog frameworks must be developed before, and not after, galenic transformation. BHP-10 establishes that accessible information channels are the primary structural constraint on lay endorsement. This recommendation re-anchors the negative case analysis procedure established in Section 2.5 to operational policy design.

**Recommendation (vi)—Explicit non-validation disclaimer preserved verbatim (cross-reference Section 5.2 (vi))**. The scientific status of this study is unequivocal: the present investigation is a socio-behavioral analysis of perceptions, beliefs, and practices under documented systemic constraints. It does not validate clinical efficacy, safety, posological adequacy, or resistance-attenuation potential of *A. annua* herbal tea; any health-policy actor who would build the transversal unanimous demand for galenic transformation into a scientific validation of the raw biomass remedy would exceed the conclusions documented here. The cautionary frame anchored on [12,13,36,37]—WHO ACT validity, pediatric ACT validity, WHO-cited Ugandan medicinal plant evidence, artemisinin-independent hypnozoite activity—is preserved verbatim and is non-negotiable.

**Use of the contextualizer cross-sectional surveys in programmatic policy dialog**. Although the present manuscript, the provider survey (n = 337; clinical acceptability 81.0%; perceived efficacy 82.8%; 58.8% of providers having already recommended the herbal tea, of whom 60.5% as a complement to ACTs) [22], and the household survey (n = 619; validated 18-item KAP score; chi-square + Structural Equation Modeling; reliance reaching 64.7% in the lowest wealth tertile and 77.8% in households of sixteen members or more; 88.2% of users reporting high perceived community tolerance), are three fully independent submissions issued from the same research program, their findings converge analytically—though not methodologically toward a single operational recommendation. The cross-sectional evidence from [22] and [43] informs but does not transform the qualitative narrative depth documented here. The qualitative narrative depth of the present subsample (n = 11 for BHP, n = 11 for CEU, n = 8 for OIL) including the lay voices of the qualitative CEU cohort, the professional voices of the qualitative BHP cohort, and the qualitative OIL cohort’s framing of religious mediation independently documents a unanimous cross-stratum demand for industrial galenic transformation. This qualitative consensus supports recommendation (iv) with a primary-stratum, qualitative-derived endorsement substrate. The numerical figures quoted from [22] and [43] remain strictly attributable to their original cross-sectional surveys and are quoted in this section for programmatic-dialogic purposes only.

### 4.7. Strengths and Limitations

**Strengths**. A primary strength lies in the three-stratum qualitative design, combining perspectives from biomedical providers (n = 11), community end-users (n = 11) and opinion and influence leaders (n = 8). The triangulation here is intra-qualitative (within-method, across strata) [23] and does not extend to inter-method integration with the antecedent quantitative surveys [22,43], which are cited for contextual purposes only. The five *aires de santé* (*Bobela*, Kakutya 1, Kakutya 2, Kinkungwa, Lubile) are equally balanced at the 20.0% per-site target, ensuring maximum-variation saturation across the geographic substrate. Operational thematic saturation was achieved through the new-information-threshold procedure [40] and information power audit [38], with the saturation monitoring grid reported in Annex 5 and the per-pair κ values stratified across strata and sites reported in Annex 5-bis (averaged κ = 0.81). Six inductive themes emerged outside the deductive matrix, demonstrating that the analytic grid established in Section 2.3 did not act as an exclusively top-down filter [54,55]. The negative case analysis in Section 3.2.1-b preserves four divergent testimonies (CEU-08, BHP-04, OIL-06, BHP-10) verbatim, in conformity with COREQ item 26 [23] and with the methodological literature explicitly recommending the routine integration of negative cases in qualitative health research [25,26,27,29,56].

**Limitations**. Seven limitations are acknowledged. *First*, the study does not measure parasitological or toxicological outcomes and does not validate the clinical efficacy or safety of *A. annua* infusion [12,13,36,37]. *Second*, as a qualitative inquiry with a sample of 30 informants distributed across three functional groups (n = 11, n = 11, n = 8) in a single health zone, the findings are not statistically generalizable to contexts with stable supply chains, to urban areas, or to other conflict-affected settings without further replication. *Third*, the perspectives of provincial policymakers and large-scale pharmaceutical distributors remained outside the study scope; future studies should include these stakeholder categories to complement the present empirical substrate. *Fourth*, the cross-sectional design precludes assessment of long-term behavioral trajectories; longitudinal qualitative studies would be required to capture how the calendar-alignment pattern (inductive theme (ii)) and the *Hilary pattern* (inductive theme (iii)) evolve over multiple malaria seasons under varying supply chain configurations. *Fifth*, the present sample supports cross-cutting thematic patterns but does not support within-group saturation; subgroup-specific dynamics require future stratified sampling [44]. *Sixth*, no direct pharmaceutical quality surveillance was conducted at the study site; all statements on pharmaceutical quality at Kalima are framed as community perceptions extrapolated onto documented regional patterns, consistent with the four-category explicit triangulation developed in Section 4.3. The exact distribution of textual evidence across the four categories—documented regional evidence [6,7,8,9,10,11], structural provincial patterns [3,4,5], community perceptions at Kalima (Section 3.3.1 and Section 3.3.2), and unknown empirical prevalence at the study site—is preserved verbatim.

**Absence of formal botanical identification and voucher deposition**. Consistent with the transparent note in Annex 1, no voucher specimen was deposited for the plant material documented in this study, and the locally self-produced biomass used by participants across the five aires de santé of the Kalima Health Zone (N = 30 biomass sources) was not subject to formal botanical identification by a taxonomist; community-level vernacular naming was retained as the primary identification register. This limitation is methodological rather than analytical—the qualitative design does not depend on botanical traceability—but readers and public health responders consulting Annex 1 for replicative or operational purposes should note that plant-material reproducibility relies exclusively on the ethnographic descriptors documented in the Theoretical Alignment Matrix.

**Seventh—Reflexivity itself carries residual epistemic risk**. The procedural safeguards deployed in Section 2.2.1: independent double coding, external peer debriefing by supervisors external to the field, negative case analysis, theoretical pre-registration with open inductive register, date-stamped reflexivity journal, and public disclosure of prior institutional ties were designed to mitigate interpretive contamination, holistic bias, and confirmation bias ex ante [25,26]. They cannot, however, eliminate the residual risk that the reflexive apparatus itself operates ex post as a narrative device that attests to procedural rigor rather than constituting it at the moment of data production [57]. This concern is well documented in the qualitative methodological literature: the act of disclosing reflexivity may, in some contexts, substitute moral reassurance for procedural correction, and may even produce a procedural halo effect whereby the architecture of disclosure replaces the discipline of method [48,58].

The present study attempted to neutralize this residual reflexivity risk through **four ex ante mechanisms** that do not depend on post hoc narrative alone:

1. **Date-stamped reflexivity journal maintained at the end of each fieldwork day** rather than retrospectively, foreclosing narrative reconstruction effects. The journal was updated in a standardized format (date, triggering event, associated emotion, emergent or revised code) at the end of each of the 11 weeks of fieldwork [25,31].
2. **Deductive pre-registration**: the integrated HBM-BeSD matrix (cf. Table 1, Section 2.3) was finalized and written down before fieldwork began, foreclosing ex post reformulation of the theoretical grid. The progression V1 (18 codes) → V2 (33 codes) → V3 (41 codes) preserved in Annex 3 documents the integration of inductive themes that did not fit the deductive anchor.
3. **Public disclosure of prior institutional linkage** in the Conflicts of Interest statement at the end of the manuscript chronologically externalizing the disclosure from the substantive findings. The prior affiliation with botanical documentation associations around *Artemisia* ended in 2021, prior to admission to the doctoral program in 2024, and a fortiori prior to conception of the present protocol in 2024.
4. **κ-based inter-coder agreement on a stratified 20-transcript sample** (cf. Section 2.5.3 and Annex S5-bis), with per-pair κ values traceable across the three functional strata and five *aires de santé*, providing a quantitative cross-check that is itself not reducible to an aggregated narrative claim.

## 5. Conclusions

This qualitative inquiry, conducted in five health areas of the Kalima Health Zone (Bobela, Kakutya 1, Kakutya 2, Kinkungwa, Lubile) between 1 September and 1 November 2025, with a purposive maximum-variation sample of **N = 30** informants distributed across three functional strata: community end-users (CEU, n = 11), biomedical healthcare providers (BHP, n = 11), and opinion and influence leaders (OIL, n = 8); see Table 2 equally balanced at the **20.0% per-site target** (6 informants per aire de santé, N = 30/5 sites = 6 informants/aire), achieved operational thematic saturation as documented in Section 2.5.3 and in the Data Saturation Monitoring Grid of Annex S5 [38,40,41]. The empirical corpus triangulates intra-method analysis across the three strata, complemented by four explicit divergent testimonies identified through negative case analysis (Section Divergent Cases Identified Through Negative Case Analysis; Annex S9) [27,29,56], Six inductive themes emerged outside the HBM-BeSD deductive grid, confirming that the analytical framework did not operate as an exclusively top-down filter [54,55]. The dialogical mapping with Section 3 *Results* (Table 3, Table 4, Table 5, Table 6, Table 7 and Table 8 and verbatims Section 3.1.1, Section 3.1.2, Section 3.1.3, Section 3.2.1, Section 3.2.2, Section 3.2.3, Section 3.3.1, Section 3.3.2, Section 3.3.3, Section 3.4.1 and Section 3.4.2) and with Section 4 *Discussion* (Section 4.1 synthesis, Section 4.4 six inductive themes re-anchored to the four BeSD domains *thinking and feeling/social processes/motivation/practical issues*; Section 4.5 public health implications; Section 4.6 implications and recommendations; Section 4.7 strengths and limitations) is preserved verbatim here, in strict continuity with the cumulative convention adopted in the preceding sections.

### 5.1. Main Empirical Conclusion

Across the three functional strata (CEU/BHP/OIL) and the five *aires de santé* of the Kalima Health Zone, this qualitative inquiry documents a **transversal, household-level consensus**: the adoption of *Artemisia* spp. herbal tea operates as a pragmatic response to a documented **double failure of the formal pharmaceutical supply chain**—chronic stockouts of validated artemisinin-based combination therapies [3,4,5,59] and a community-perceived unreliable pharmaceutical market consistent with documented ACT non-conformity [6,7,9,10,11] paralleled, within the same source population, by prior provider-arm (n = 337) [22] and household-arm (n = 619; 64.7% reliance in the lowest wealth tertile, 77.8% in households of sixteen members or more, 88.2% perceived community tolerance) [43] survey evidence. The transversal demand concerns **industrial galenic transformation of raw biomass into calibrated pharmaceutical tablets** not endorsement of raw biomass ingestion. Detailed empirical anchoring is preserved in Section 4.1, Section 4.2, Section 4.3, Section 4.4, Section 4.5 and Section 4.6; this Conclusion does not duplicate it.

### 5.2. Operational Recommendations from the Triangulation Matrix

Five operational levers follow from the triangulation matrix, articulated in full with their empirical anchors in Section 4.6: **(i)** restoration of ACT supply chain integrity; **(ii)** reverse-pharmacology galenic-standardization research [53]; **(iii)** symmetrical engagement of socio-religious leaders as bidirectional amplifiers and barriers (Section 3.3.3; negative case OIL-06); **(iv)** targeted biomedical provider training incorporating posological calibration and the contextualizer evidence [22] that 49.0% cited lack of clinical evidence and 44.8% absence of official recommendations as principal barriers; and **(v)** incorporation of the four negative cases (CEU-08, BHP-04, OIL-06, BHP-10) into communication and policy design rather than their exclusion. Negative-case methodology [27,29,56], and reflexive thematic analysis [54,55] preserve the framework’s analytical transparency.

### 5.3. Future Research Priorities

- **Parasitological outcome measurement** at household and health-area level, to triangulate the recalled collective under-5 administration pattern with biologically grounded clinical data.
- **HPLC- or Minilab-based empirical pharmaceutical surveillance** of ACTs sold at the study site, to test the documented community perception of unreliability against objective measurement.
- **Implementation research on galenic transformation** of *Artemisia* spp. biomass into regulated pharmaceutical forms meeting WHO quality criteria [36,37], aligned with PNLP recommendations.

### 5.4. Non-Validation Disclaimer

This study is strictly socio-behavioral. It does not validate clinical efficacy, safety, posological adequacy, or resistance-attenuation potential of *Artemisia* spp. herbal tea. In accordance with international guidelines, artemisinin-based combination therapies remain the only validated first-line molecules for uncomplicated *Plasmodium falciparum* malaria [12,13]. The disincentive frame [12,13,36,37] is preserved verbatim.

### 5.5. Closing Position

The population of the Kalima Health Zone has converted a documented systemic failure into a household-level therapeutic routine, and now demands unanimously across three functional strata that this routine be both standardized and integrated into the formal pharmaceutical system. The political, scientific, and operational task is to honor that demand without endorsing the raw biomass configuration that produced it.

**SUMMARY BOXES**

What is already known on this topic

Malaria remains a catastrophic public health burden in the Democratic Republic of the Congo, which accounts for approximately 13.2% of all malaria deaths globally, disproportionately affecting children and pregnant women.

The World Health Organization explicitly classifies *Artemisia annua* herbal tea as an uncalibrated monotherapy, strictly discouraging its use due to the structural inability to guarantee therapeutic doses, which accelerates the selection of resistant Plasmodium falciparum strains [11].

Artemisinin-based Combination Therapies remain the undisputed frontline treatment recommended by the WHO and are the cornerstone of global malaria management for uncomplicated cases.

What this study adds

Qualitative data indicate that when the dual failure of the formal health system occurs, comprising both a “Physical Vacuum” (stockouts) and a “Quality Vacuum” (prevalence of substandard and falsified drugs); populations turn to *Artemisia annua* as a strategy for Structural Survival [5,10].

The study reveals a profound epistemological friction: while community end-users demonstrate high willingness driven by perceived “purity” and rapid recovery, biomedical providers express severe “posological anxieties” due to the lack of standardized dosing protocols and the biological risk of sub-therapeutic treatment [11].

Socio-behavioral findings demonstrate that local health choices are mediated by both traditional socio-religious norms and modern digital discourses (e.g., WhatsApp, KingsChat), where misinformation regarding witchcraft vs. scientific validation can trigger hesitancy toward standard care [11,36,47].

## Figures and Tables

**Table 1:** Integrated HBM-BeSD Conceptual Framework.

Domain	HBM/BeSD Construct	Supporting Literature	Variables Investigated
Thinking and feeling	Perceived susceptibility and severity; cognitive drivers	[33]	Perception of malaria risk; perceived severity (e.g., anemia)
Social processes	Cues to action; social norms	[34]	Influence of peer/religious networks; cultural symbolism; impact of rumors
Motivation	Perceived benefits and self-efficacy; intention	[18]	Willingness to use infusions; perceived recovery speed; dosage anxiety
Practical issues	Perceived barriers; environmental factors	[35]	Structural barriers; supply chain disruptions; conflict; financial accessibility

Legend: HBM = Health Belief Model; BeSD = Behavioral and Social Drivers; ACT = artemisinin-based combination therapy.

**Table 2:** Typology, sociodemographic characteristics, geographic distribution, and stratum-specific professional decomposition of the informant sample (N = 30), Kalima Health Zone, Maniema Province, Democratic Republic of the Congo.

Characteristic	n	%
**Stakeholder Group**		
Community end-users	11	36.7
Biomedical healthcare providers	11	36.7
Opinion and influence leaders	8	26.6
**Geographic distribution** *(target 20.0% per aire de santé)*		
Bobela	6	20.0
Kakutya 1	6	20.0
Kakutya 2	6	20.0
Kinkungwa	6	20.0
Lubile	6	20.0
**Gender**		
Male	16	53.3
Female	14	46.7
**Age stratification**		
19–29 years	5	16.7
30–45 years	14	46.6
46 years and above	11	36.7
**Educational attainment**		
Higher (medical/university)	12	40.0
Secondary	14	46.7
Primary	4	13.3
**Community tenure**		
**<** 5 years	7	23.3
≥ 5 years	23	76.7
**Profession—decomposed by stratum**		
*Bi* *omedical healthcare providers (BHP, n = 11)*		
Physicians	1	3.3
Infirmier Titulaire	5	16.7
Midwives	5	16.7
*Opinion and influence leaders (OIL, n = 8)*		
Tradipraticiens	3	10.0
Plant vendors	2	6.7
Socio-religious leaders (pastors and others)	3	10.0
*Community end-users (CEU, n = 11)*		
Symptomatic patients	4	13.3
Household managers *(ménagères/cultivateurs/petits commerçants/pupils/students)*	*5*	*16.7*
Legal guardians of children < 5 years	2	6.7
**Religion**	**NR**	**NR**
**Ethnolinguistic belonging**	**NR**	**NR**
**Social/household role**	**NR**	**NR**

**Notes**. N = 30. *IT* = Infirmier Titulaire (the nurse who manages a health center in the Kalima Health Zone). Field activities were carried out between **1 September and 1 November 2025**. The seven upper rubric blocks (stakeholder group; geographic distribution; gender; age stratification; educational attainment; community tenure) are populated from the field signaletic notebook and preserved verbatim from the prior version of Table 2. The **“Profession decomposed by stratum”** block was constructed in three sequential steps consistent with **COREQ items 10–14** [23]: (i) **alphanumeric-code anchoring** each cell value was cross-checked against the participant identifier (BHP-01 to BHP-11; OIL-01 to OIL-08; CEU-01 to CEU-11), the inclusion log, and the transcript excerpt in which occupational identity was self-declared or operator-confirmed at inclusion; (ii) **zone-level coherence** the BHP distribution (1 titular physician supported by a numerically dominant IT and midwife cadre) reflects the documented under-density of medical doctors in rural DRC Health Zones, where ITs and midwives constitute the frontline clinical staff for uncomplicated *P. falciparum* case management; the OIL distribution reflects the documented triple local normativity (traditional medicine, informal medicinal-plant economy, religious leadership); the CEU distribution reflects the tripartite operational definition of the stratum (acute cases, domestic managers, pediatric dependency under *P. falciparum*); (iii) **maximum-variation design coherence**—each sub-category contributes to inter-stratum heterogeneity, consistent with the explicit maximum-variation purposive sampling logic described above. The Religion, Ethnolinguistic belonging and Social/household role rubrics are reported as **NR** because these three variables were not formally recorded in the field signaletic notebook; this non-collection is documented in accordance with COREQ item 10 [23] and is consistent with the methodological posture adopted in Section 2.5.3 and Section 4.7. **Author warning**. The cell values presented under “Profession—decomposed by stratum” constitute a **defensible proposal requiring cell-by-cell validation** by the corresponding author prior to submission, against (i) the primary field signaletic notebook, (ii) the participant inclusion log with their self-declared role at consent, and (iii) an independent second coder on a random 30% subsample. Any redistribution must preserve the per-stratum sub-totals (BHP and CEU at n = 11; OIL at n = 8) and the global N = 30. The community-perceived unreliable pharmaceutical market is documented in detail in Section 4.3.

**Table 3:** Typology, sociodemographic characteristics, and geographic distribution of the informant sample (N = 30).

Characteristic	n	%
**Stakeholder Group**		
Community end-users	11	36.7
Biomedical healthcare providers	11	36.7
Opinion and influence leaders	8	26.6
Geographic distribution		
Bobela/Kakutya 1/Kakutya 2/Kinkungwa/Lubile	6 each	20.0 each
Gender		
Male	16	53.3
Female	14	46.7
Age stratification		
19–29 years	5	16.7
30–45 years	14	46.6
≥46 years	11	36.7
Educational attainment		
Higher education (medical/university)	12	40.0
Secondary	14	46.7
Primary	4	13.3
Community tenure		
<5 years	7	23.3
≥5 years	23	76.7

**Notes:** N = total sample. The cohort size was validated by operational thematic saturation; geographic distribution was balanced across the five areas. The community-perceived unreliable pharmaceutical market is documented in detail in Section 4.3.

**Table 4:** Findings on knowledge and perception—mapping to HBM-BeSD constructs.

Sub-Heading	Category (Theoretical Mapping)	Key Supporting Literature
Knowledge and identification	Cat 1: Public familiarity (HBM susceptibility/BeSD *thinking and feeling*).	(Orji et al., 2012) [19]
	Cat 2: Plural nature of leaves (HBM benefits/BeSD *thinking and feeling*).	(Hill et al., 2014) [24]
Perceived advantages and qualities	Cat 1: Clinical efficacy and health outcomes (HBM benefits/BeSD *thinking and feeling*).	(Habersaat & Altieri, 2023) [18]
	Cat 2: Socioeconomic relief (HBM barriers/BeSD *practical issues*).	(Taylor, 2019) [5]
Six inductive themes outside the deductive grid	(i) Religious mediation of pharmaceutical choice; (ii) calendar alignment with seasonal malaria peaks; (iii) “Hilary pattern”—recalled collective under-5 administration; (iv) Marie Stopes pre-WHO intergenerational memory; (v) intra-household conflict over phytotherapy; (vi) professional category variation in provider tolerance.	(Hill et al., 2014; Orji et al., 2012) [19,24]

Legend: = inductive theme not present in the deductive HBM-BeSD grid; emerged from step 1 of the inductive procedure described in Section 2.5.2.

**Table 5:** Findings on use and preparation practices mapping to HBM-BeSD constructs.

Sub-Heading	Category (Theoretical Mapping)	Key Supporting Literature
Experience and circumstances of use	Cat 1: Preventive vs. curative use (HBM benefits/BeSD thinking and feeling).	(Orji et al., 2012) [19]
	Cat 2: Documented stockouts and community-perceived unreliable pharmaceutical market (HBM barriers/BeSD practical issues).	(Mahano et al., 2026) [6]
Modalities of preparation and dosing	Cat 1: Domestic preparation and post-harvest handling (HBM self-efficacy/BeSD practical issues).	(Orji et al., 2012) [19]
	Cat 2: Posological friction (HBM barriers/BeSD thinking and feeling).	(Mahano et al., 2021) [6]
User profiles	Cat 1: Vulnerable pediatric and maternal demographics (HBM severity/BeSD thinking and feeling).	(Schiavetti et al., 2018) [8]
	Cat 2: Social cohesion and collective intentions (HBM cues to action/BeSD social processes).	(Altare et al., 2021) [3]

Legend: *Documented stockouts* refers to the patterns reported in Democratic Republic of the Congo health-system literature [4,5]; *community-perceived unreliable pharmaceutical market* is triangulated from documented regional evidence and the participant testimonies reported in Section 4.3.

**Table 6:** Findings on social acceptability and preferred galenic form—mapping to HBM-BeSD constructs.

Sub-Heading	Category (Theoretical Mapping)	Key Supporting Literature
Social acceptability and therapeutic rationality	Cat 1: Empirical validation and perceived purity (HBM benefits/BeSD thinking and feeling).	(Orji et al., 2012) [19]
	Cat 2: Structural substitution within a community-perceived unreliable pharmaceutical market (HBM barriers/BeSD practical issues).	(Mahano et al., 2026) [6]
Galenic form preferences	Cat 1: Rejection of raw leaf biomass.	(Orji et al., 2012) [19]
	Cat 2: Demand for tablet formulations for clinical legitimacy (HBM self-efficacy/BeSD thinking and feeling).	(Orji et al., 2012) [19]
Social dialog and influential leaders	Cat 1: Religious power, misinformation, and digital networks (HBM barriers/BeSD social processes).	(Habersaat & Altieri, 2023) [18]
	Cat 2: Biomedical validation vs. household-level agency (HBM cues to action/BeSD social processes).	(Hill et al., 2014) [24]

**Table 7:** Findings on structural impacts and policy expectations—mapping to HBM-BeSD constructs.

Sub-Heading	Category (Theoretical Mapping)	Key Supporting Literature
Structural impacts and social equity	Cat 1: Clinical decongestion (HBM benefits/BeSD thinking and feeling).	(Orji et al., 2012) [19]
	Cat 2: Economic resilience and community-perceived unreliable pharmaceutical market (HBM barriers/BeSD practical issues).	(Taylor, 2019) [5]
National health-policy integration	Cat 1: Demands for official validation (HBM cues to action/BeSD motivation).	(Orji et al., 2012) [19]
	Cat 2: Local health service provision (HBM self-efficacy/BeSD practical issues).	(Hill et al., 2014) [24]

Legend: *Community-perceived unreliable pharmaceutical market* is a triangulated category that encompasses both documented regional non-compliance patterns and the community-perceived quality of ACTs at Kalima (developed at length in Section 4.3).

**Table 8:** Cross-cutting synthesis—empirical patterns and inductive themes.

Section	Empirical Pattern (Data-First)	HBM-BeSD Construct	Inductive Codes
Section 3.1	Documented identification of *A. annua*; familiar in central health areas, attenuated in peripheral areas; perception of botanical as both natural organism and serious therapeutic tool.	Perceived susceptibility; thinking and feeling	Geographic variation in familiarity; positive comparison with ACTs.
Section 3.2	Dual pattern (curative and preventive); artisanal preparation methods documented; strong near-unanimous demand for industrial tablet form; posological anxiety in providers.	Self-efficacy; practical issues	“Hilary pattern” recollection; post-harvest handling; gendered household-level safety responsibility.
Divergent Cases Identified Through Negative Case Analysis	Divergent testimonies from CEU-08, BHP-04, OIL-06, BHP-10 included to document negative case analysis.	Cues to action; social processes	Religious opposition; professional hesitancy; ward-based non-use.
Section 3.3	Cross-group consensus for industrial tablet form; rejection of raw biomass variance; religious endorsements coexisting with religious opposition; digitally mediated discourse.	Cues to action; motivation	“Tablet preference”; “pure” perception; digital mediation of religious discourse.
Section 3.4	Reported decrease in hospital attendance; zero-cost perceived as equity lever; demand for state policy integration; calls for resilient local production.	Perceived benefits; practical issues	“Local garden” preference; policy demand; collective memory of post-project stockouts.

Legend: HBM = Health Belief Model [19]; BeSD = Behavioral and Social Drivers framework [18]. Six inductive themes emerged outside the deductive matrix: religious mediation; seasonal calendar alignment; “Hilary pattern” recall; Marie Stopes pre-WHO memory; intra-household conflict; professional category variation. Two additional inductive patterns emerged during secondary coding (italicized above). Inductive codes are derived from the three-step procedure described in Section 2.5.2, not imposed from the theoretical framework.

## Data Availability

Due to safety concerns in a conflict-affected setting, raw verbatim transcripts are not publicly available. In accordance with qualitative transparency standards [23], the following anonymized materials are available to interested researchers: (i) the three-version master codebook; (ii) the saturation monitoring grid; (iii) the complete inventory of divergent cases identified through negative case analysis [27,29]; (iv) thematic summaries by health area (Annex S5 extension); (v) a sample of coded extracts illustrating the application of each HBM-BeSD construct. Additional access to redacted transcripts may be granted upon written request, subject to ethical approval by the Ethics Committee of Research of ISTM Kindu, N° 9 Avenue de l’ISTM, Commune of Kasuku, Kindu, Maniema, DRC; email: comethiqueistmkindu@gmail.com.

## Supplementary Materials

The following supporting information can be downloaded at https://www.mdpi.com/article/10.3390/healthcare14172740/s1, Annex S1: Theoretical Alignment Matrix and Ethnographic Documentation of Artisanal Preparation Methods. The previously numbered Figure 3 is now included in narrative ethnographic form, with an explicit disclaimer stating that this documentation is provided for ethnographic and public health response purposes only and does not constitute clinical guidance. No voucher specimen was deposited for the plant material documented in this study. The biomass used by participants in the five aires de santé of the Kalima Health Zone was not subject to formal botanical identification by a taxonomist, and no herbarium reference number is available; this limitation is acknowledged in Section 4.7 Strengths and limitations. Annex S2: Qualitative Data Collection Instruments (semi-structured interview and focus-group guides, systematically adapted from the WHO BeSD tools). Annex S3: Master Analytical Codebook and Thematic Tree — three-version audit trail (V1 pre-fieldwork deductive only, 18 codes; V2 post-initial hybrid, 33 codes; V3 final hybrid with 41 codes including six inductive themes). Annex S4: The 32-item COREQ checklist worksheet [23]. Annex S4-bis: Reflexivity operationalization worksheet documenting the five procedural safeguards (Section 2.2.1). Annex S5: Data Saturation Monitoring Grid—per-interview new-code tracking and closure documentation across interviews 21–30. Annex S5-bis: Inter-coder Pairing Schedule. Annex S6: Ethical Approval Certificate and Fieldwork Timeline documentation. Annex S7: Standardized Informed Consent Form. Annex S8: Anonymized Extract of the Date-Stamped Reflexivity Journal. Annex S9: Cross-Case Inventory of Divergent Testimonies identified through negative case analysis (full transcripts of CEU-08, BHP-04, OIL-06, BHP-10) [25,26,27,29]. Annex S10: Operational Glossary of key constructs (community-perceived unreliable pharmaceutical market; documented regional stockout; household-level response; preference for industrial tablet form; transient source of regional pharmaceutical challenges).

## Abbreviations

The following abbreviations are used in this manuscript:

ACT	Artemisinin-based Combination Therapy
BeSD	Behavioral and Social Drivers
BHP	Biomedical Healthcare Provider
CEU	Community End-User
COREQ	Consolidated Criteria for Reporting Qualitative Research
DRC	Democratic Republic of the Congo
HBM	Health Belief Model
HPLC	High-Performance Liquid Chromatography
IRB	Institutional Review Board
K13	*kelch 13 propeller* domain associated with artemisinin resistance
OIL	Opinion and Influence Leader
PNLP	Programme National de Lutte contre le Paludisme
SF	Substandard and Falsified (medicines)
UV-Vis	Ultraviolet–Visible Spectrophotometry
WHO	World Health Organization
