# Supplementary material for: Perceptions and Acceptability of Artemisia annua Herbal Tea for Malaria Treatment: A Qualitative Study in Kalima Health Zone, Maniema, Democratic Republic of the Congo
Source: Healthcare (Basel). 2026 Aug 27;14(17):2740. doi: 10.3390/healthcare14172740 (PMC13565392; doi:10.3390/healthcare14172740)
Supplement: Supplementary file 1 [file healthcare-14-02740-s001.zip › healthcare-4362638-supplementary.pdf]

ANNEXE S1

Théoretical Alignment Matrix and Ethnographic Documentation of Artisanal Preparation Methods

Supplementary Materials to "Perceptions and Acceptability of Artemisia annua Herbal Tea for Malaria Treatment: A Qualitative Study in Kalima Health Zone, Maniema, Democratic Republic of the Congo"

Preamble to Annex S1

This annex documents the operational alignment of the in-depth qualitative inquiry with the integrated Health Belief Model and the WHO Behavioral and Social Drivers framework. It serves three distinct documentary purposes that the referee and the policy reader can consult independently of the main text:

1. **Part A Theoretical Alignment Matrix.** A four-column matrix mapping each HBM construct and each BeSD dimension to its theoretical dimension, its corresponding interview/FGD probe, and the V3 (round-three) emergent codes, with full citation of the methodological literature underpinning the analytical architecture (Habersaat & Altieri, 2023; Orji et al., 2012; Tong et al., 2007).
2. **Part B Six Inductive Themes.** A verbatim list of the six themes that emerged outside the deductive HBM-BeSD matrix during hybrid thematic analysis (Byrne, 2021; Campbell et al., 2021), with interpretive caveats for two of them.
3. **Part C Ethnographic Documentation of Artisanal Preparation Methods.** A verbatim selection of testimonies describing artisanal preparation methods reported by study participants, framed by an editorial disclaimer that documents the evidentiary literature on standardized *Artemisia annua* trials and pharmacognostic properties without extending them into clinical guidance (Angupale et al., 2023; Ashraf et al., 2021; Assefa et al., 2024; Magalhães et al., 2016; Shibeshi et al., 2021; Sinclair et al., 2009; Suberu et al., 2013; White et al., 2009).

The annex therefore complements section3.2.2 of the manuscript (preparation modalities) by making explicit the theoretical anchoring of every analytic code, the inductive structure of the qualitative argument, and the ethnographic detail underlying the policy recommendation against uncalibrated monotherapy.

Part A Theoretical Alignment Matrix (HBM × BeSD × Interview Probes × Emergent Codes)

The matrix operationalizes the integrated Health Belief Model and the WHO Behavioral and Social Drivers framework to ensure every analytical code is theory-anchored and probed through a corresponding interview item, following COREQ Domain 2 *Consolidated criteria for reporting qualitative research: a 32-item checklist for interviews and focus groups*(Tong et al., 2007) and the framework construction principles in *Behavioural Sciences for Better Health: WHO Resolution and Action Framework*(Habersaat & Altieri, 2023) and *Towards an Effective Health Interventions Design: An Extension of the Health Belief Model*(Orji et al., 2012).

HBM/BeSD Construct Domain	Theoretical Dimension	Illustrative Probe in Interview/FGD Guide	Emergent V3 Codes
**Thinking & feeling**	Perceived susceptibility	"How do you perceive the risk of contracting malaria in your current environment?"	`hyper-endemicity`; `continuous vector exposure` *The "empty void" is a crowded space: health service provision at the margins of fragile and conflict affected states*(Hill et al., 2014); *Descriptive ethnobotanical studies are needed for the rescue operation of documenting traditional knowledge*(Łuczaj, 2023); *From Insecurity to Health Service Delivery: Pathways and System Response Strategies in the Eastern Democratic Republic of the Congo*(Altare et al., 2021)
**Thinking & feeling**	Perceived severity	"What are the consequences when modern antimalarial drugs are unavailable due to conflict or stockouts?"	`supply chain collapse`; `armed conflict disruption`; `lethal risk`; `pharmaceutical stockouts` *The tyranny of empty shelves: Scarcity and the political manufacture of antiretroviral stock-outs in South Kivu, the Democratic Republic of the Congo*(Taylor, 2019); *Systems Thinking for Supply Chains: Identifying Bottlenecks Using Process Mapping of a Child Health Intervention in the Democratic Republic of the Congo*(Karim et al., 2024)
**Thinking & feeling**	Perceived benefits (therapeutic)	"In your experience, what are the main advantages of using *A. annua* infusion compared to other treatments?"	`rapid clinical recovery`; `anemia correction`; `financial sovereignty`; `cost-barrier removal` *Artemisia annua: A New Version of a Traditional Tea under Randomized, Controlled Clinical Trial for the Treatment of Malaria*(Magalhães et al., 2016); *Anti-Plasmodial Polyvalent Interactions in Artemisia annua L. Aqueous Extract – Possible Synergistic and Resistance Mechanisms*(Suberu et al., 2013); *Public Health Perspectives on Integrating Artemisia annua Tea for Uncomplicated Malaria Treatment: A Cross-Sectional Study of Perceptions and Acceptability among*

			Healthcare Workers in Kalima District, Maniema, DRC(Munyangi wa Nkola & Camara, 2026)
**Thinking & feeling**	Perceived benefits (egalitarian)	"Does this remedy favor those who cannot afford ACTs?"	zero-cost access; household financial sovereignty
**Thinking & feeling**	Perceived barriers — posological	"What concerns do you have regarding dosage of the herbal tea?"	dosing anxiety; raw leaf rejection; pfk13 selection fear *Emergence of Plasmodium falciparum strains with artemisinin partial resistance in East Africa and the Horn of Africa: is there a need to panic?*(Assefa et al., 2024); *Hyperparasitaemia and low dosing are an important source of anti-malarial drug resistance*(White et al., 2009); *Country wide surveillance reveals prevalent artemisinin partial resistance mutations with evidence for multiple origins and expansion of high level sulfadoxine-pyrimethamine resistance mutations in northwest Tanzania*(Juliano et al., 2023)
**Thinking & feeling**	Perceived barriers — socio-cultural	"Are there cultural beliefs that influence whether this remedy is used?"	witchcraft/religious rumors *Harnessing traditional healers role in strengthening Africa's public health response: A case study of Mpox outbreak*(Osborne et al., 2025); *Malaria vaccine efficacy, safety, and community perception in Africa: a scoping review of recent empirical studies*(Chutiyami et al., 2024)
**Social processes**	Cues to action	"Who or what influenced your first decision to use or recommend *A. annua*?"	NGO/institutional pathways; pastoral influence; medical leadership validation *Public Health Perspectives on Integrating Artemisia annua Tea for Uncomplicated Malaria Treatment: A Cross-Sectional Study of Perceptions and Acceptability among Healthcare Workers in Kalima District, Maniema, DRC*(Munyangi wa Nkola & Camara, 2026); *Towards an Effective Health*

			Interventions Design: An Extension of the Health Belief Model(Orji et al., 2012); *Behavioural Sciences for Better Health: WHO Resolution and Action Framework*(Habersaat & Altieri, 2023)
**Social processes**	Social norms & collective memory	"What do elders or religious leaders say about this remedy?"	`community endorsement`; `collective memory of post-project stockouts` *From Insecurity to Health Service Delivery: Pathways and System Response Strategies in the Eastern Democratic Republic of the Congo*(Altare et al., 2021); *The "empty void" is a crowded space: health service provision at the margins of fragile and conflict affected states*(Hill et al., 2014)
**Motivation**	Self-efficacy (preparation)	"How confident do you feel preparing the infusion at home?"	`artisanal mastery`; `boiling mechanics`; `gendered pot management` *Artemisia annua: A New Version of a Traditional Tea under Randomized, Controlled Clinical Trial for the Treatment of Malaria*(Magalhães et al., 2016)
**Motivation**	Self-efficacy (cure trajectory)	"Would you give this remedy to a sick child under five?"	`maternal confidence`; `household-level agency` *Malaria profile and socioeconomic predictors among under-five children: an analysis of 11 sub-Saharan African countries*(Anjorin et al., 2023); *The Quality of Medicines Used in Children and Supplied by Private Pharmaceutical Wholesalers in Kinshasa, Democratic Republic of Congo: A Prospective Survey*(Schiavetti et al., 2018)
**Motivation**	Willingness / future intention	"Would you adopt *A. annua* if it were an industrially produced tablet?"	`tablet preference`; `industrial galenic preference` (inductive) *Public Health Perspectives on Integrating Artemisia annua Tea for Uncomplicated Malaria Treatment: A Cross-Sectional Study of Perceptions and Acceptability among Healthcare Workers in Kalima District, Maniema, DRC*(Munyangi wa Nkola & Camara, 2026); *Towards an Effective Health Interventions Design: An Extension of the*

			Health Belief Model(Orji et al., 2012); *Behavioural Sciences for Better Health: WHO Resolution and Action Framework*(Habersaat & Altieri, 2023)
**Practical issues**	Structural barriers geography	"How does geographic isolation affect your access to ACTs?"	`geographical isolation`; `transportation cost`; `seasonal road blockage`; `enclavement` *The tyranny of empty shelves: Scarcity and the political manufacture of antiretroviral stock-outs in South Kivu, the Democratic Republic of the Congo*(Taylor, 2019); *Systems Thinking for Supply Chains: Identifying Bottlenecks Using Process Mapping of a Child Health Intervention in the Democratic Republic of the Congo*(Karim et al., 2024)
**Practical issues**	Pharmaceutical quality perception (community-perceived)	"How confident are you in the quality of ACTs you find in your local market?"	`community-perceived unreliable pharmaceutical market` (triangulated category) *Pharmaceutical quality of antimalarial drugs: quinine sulfate and Artemether/Lumefantrine tablets sold on Bukavu Market*(Mahano et al., 2021); *Assessment of the Quality of Antimalarial Medicines Sold in Bukavu, DRC*(Mahano et al., 2026); *Quality of the antimalarial medicine artemether – lumefantrine in 8 cities of the Democratic Republic of the Congo*(Mufusama et al., 2018); *The Quality of Medicines Used in Children and Supplied by Private Pharmaceutical Wholesalers in Kinshasa, Democratic Republic of Congo: A Prospective Survey*(Schiavetti et al., 2018); *Prevention, Detection and Response to incidences of substandard and falsified medical products in the Member States of the Southern African Development Community*(Kniazkov et al., 2020); *Detection of Substandard and Falsified Antibiotics Sold in the Democratic Republic of the Congo Using Validated HPLC and UV-Visible Spectrophotometric Methods*(Tshilumba et al., 2023)
**Practical issues**	Calendar / seasonal alignment	"Does malaria frequency	`seasonal calendar alignment` (inductive)

follow specific
seasonal
cycles?"

Part B Six Inductive Themes

These themes emerged outside the deductive HBM-BeSD matrix and are reported verbatim from the manuscript as stated in Table 3, following established reflexive thematic analysis methodology *Reflexive Thematic Analysis for Applied Qualitative Health Research*(Campbell et al., 2021) and *A worked example of Braun and Clarke's approach to reflexive thematic analysis*(Byrne, 2021):

1. **Religious mediation of pharmaceutical choice**
2. **Calendar alignment with seasonal malaria peaks**
3. **"Hilary pattern" recalled collective under-5 administration** *(interpretive caveat flagged in §2.5.2: this theme should be read with caution, since it relies on retrospective recall bias in a medicine-context where caregivers frequently aggregate their children's febrile episodes under a single named host project/programme; the term is reproduced as cited by participants and is not endorsed by the authors as an objective pathology entity)*
4. **Marie Stopes pre-WHO intergenerational memory** *(interpretive caveat flagged in §2.5.2: this theme documents a lay genealogy of A. annua use predating formal WHO endorsement of ACTs; it should not be confused with formal pharmacopoeia validation; the institutional eponym is reproduced verbatim as used by participants)*
5. **Intra-household conflict over phytotherapy**
6. **Professional category variation in provider tolerance**

Part C Ethnographic Documentation of Artisanal Preparation Methods

Editorial disclaimer. The following narrative describes preparation methods reported by study participants verbatim in §3.2.2 of the manuscript, supplemented by the documented evidentiary literature on related standardized trials *Artemisia annua: A New Version of a Traditional Tea under Randomized, Controlled Clinical Trial for the Treatment of Malaria*(Magalhães et al., 2016) and pharmacognostic properties *Anti-Plasmodial Polyvalent Interactions in Artemisia annua L. Aqueous Extract – Possible Synergistic and Resistance Mechanisms*(Suberu et al., 2013). This documentation is provided **for ethnographic and public health response purposes only and does not constitute clinical guidance**. The WHO discourages the use of unstandardized *Artemisia annua* monotherapy, including artisanal infusions, due to documented risks of sub-therapeutic dosing and the selection of artemisinin-resistant *Plasmodium falciparum* strains *A review of efficacy and safety of Ugandan anti-malarial plants with application of RITAM score*(Angupale et al., 2023); *Artemisinin-independent inhibitory activity of Artemisia sp. infusions against different Plasmodium stages including relapse-causing hypnozoites*(Ashraf et al., 2021); *Emergence of Plasmodium falciparum strains with artemisinin partial resistance in East Africa and the Horn of Africa: is there a need to panic?*(Assefa et al., 2024); ACTs remain the validated first-line treatment *Efficacy and safety of artemisinin-based*

combination therapies for the treatment of uncomplicated malaria in pediatrics: a systematic review and meta-analysis(Shibeshi et al., 2021) and *Artemisinin-based combination therapy for treating uncomplicated malaria*(Sinclair et al., 2009). Describing how a household prepares an infusion does not validate the clinical efficacy or safety of that preparation.

Modality A —Fresh-leaf boiling (verbatim from BHP-07 and CEU-06 testimonies, §3.2.2)

> *"For me, here is the exact post-harvest protocol: we used to consume it after harvesting it, and we always dried it strictly in the shade."* — BHP-07, Bobela Health Area

> *"Regarding Artemisia, I can describe a very basic method of preparation to you. Personally, I did nothing more than boiling it and then I drink it, that is more than enough."* — CEU-06, Kakutya 2 Health Area

This sample of verbatim preparation testimony is reproduced verbatim from §3.2.2. No additional method step or biological predictions are added. The epidemiological and methodological literature cited above *Anti-Plasmodial Polyvalent Interactions in Artemisia annua L. Aqueous Extract – Possible Synergistic and Resistance Mechanisms*(Suberu et al., 2013); *Artemisia annua: A New Version of a Traditional Tea under Randomized, Controlled Clinical Trial for the Treatment of Malaria*(Magalhães et al., 2016); *Hyperparasitaemia and low dosing are an important source of anti-malarial drug resistance*(White et al., 2009); *Emergence of Plasmodium falciparum strains with artemisinin partial resistance in East Africa and the Horn of Africa: is there a need to panic?*(Assefa et al., 2024) frames the discussion of standardization; it does not describe Kalima biomass composition **no independent HPLC-PDA analysis was conducted on Kalima-sourced biomass in this study.**

Annex S2 Qualitative Data Collection Instruments

A Common Opening Script

> Greeting. Thank you for agreeing to speak with us. There are no right or wrong answers. We are interested in your experience with malaria in your household and with the plants used here. The conversation will last 45–75 minutes. With your permission, audio-recording will take place. You may stop at any time, refuse any question, or request deletion of the recording. Anonymity is guaranteed: your name will not appear in any report; you will be identified by a functional code. This study is part of a doctoral research project at the Gamal Abdel Nasser University of Conakry. Ethical clearance: ISTM-Kindu Ref. 035/ISTM-KD/C.E.R. I/PRESI/IRBE/2025.

B Individual Semi-Structured Interview Guide Community End-Users

Probe area	Suggested probes	Theoretical mapping
**1. Public familiarity with *A. annua***	Recognition (visual); local name; first source of	HBM perceived susceptibility (Orji et al., 2012); BeSD knowledge (Habersaat &

	information	Altieri, 2023)
**2. Information channels**	Family-CEW conversations; pastor; mobile phone; project history	Cues to action (Chutiyami et al., 2024; Orji et al., 2012)
**3. Experience of use**	Curative vs preventive trajectory; alternatives tried first; abandonment periods	HBM benefits (Orji et al., 2012); practical issues (Habersaat & Altieri, 2023)
**4. Modalities of preparation**	Walk-through: fresh vs dried; boiling mechanics; dose frequency; duration of treatment	Self-efficacy (Magalhães et al., 2016; Orji et al., 2012); practical issues
**5. Perceived clinical recovery**	Time to defervescence; appetite restoration; comparison with ACT tablets	HBM benefits (Orji et al., 2012)
**6. Perceived barriers to ACTs**	Stockouts; cost; distance; observed ineffectiveness; side effects	HBM barriers; practical issues (Karim et al., 2024; Orji et al., 2012; Taylor, 2019)
**7. Religious and social context**	Role of pastor/catechist/healer; family council on therapy; objections raised	Social processes (Chutiyami et al., 2024; Habersaat & Altieri, 2023; Osborne et al., 2025)
**8. Pharmaceutical market quality perception**	How confident are you in the quality of ACTs in the local market?	Practical issues (community-perceived unreliable pharmaceutical market) (A. O. Mahano et al., 2021; A. Z. Mahano et al., 2026)
**9. Preferred galenic form**	Tablet, capsule, tea, standardized infusion: which would you trust most?	Tablet preference (inductive) (Habersaat & Altieri, 2023; Munyangi wa Nkola & Camara, 2026)
**10. Societal / household dynamics**	Who decides for a sick child? Tensions with grandmother or husband?	Intra-household conflict (inductive) (Byrne, 2021)
**11. Future intentions**	If a standardized industrial tablet existed, would you use it? Pay for it?	Willingness / future intention (Munyangi wa Nkola & Camara, 2026; Orji et al., 2012)
**12. Closing**	Have we missed something?	Member-checking

C Individual Semi-Structured Interview Guide — Biomedical Healthcare Providers

Probe area	Suggested probes	Theoretical mapping
**1. Professional identity and facility context**	Cadre; years in Kalima; monthly caseload	Descriptive
**2. ACT supply-chain reality**	Frequency of stockouts; percentage of orders fulfilled; order-to-receipt delay	HBM barriers (Orji et al., 2012); practical issues (Habersaat & Altieri, 2023; Karim et al., 2024; Taylor, 2019)
**3. Quality concerns**	Suspected counterfeit encounters; laboratory confirmation; clinical therapeutic failures with ACTs	Practical issues (Kniazkov et al., 2020; A. O. Mahano et al., 2021; A. Z. Mahano et al., 2026; Tshilumba et al., 2023)
**4. Patient disclosure of *A. annua* self-medication**	How often do patients report concurrent use? Before? During? After ACT?	Practical issues (Habersaat & Altieri, 2023)
**5. Posological anxiety**	Variable biomass concentration; risk of sub-therapeutic dosing	Resistant selection; posological anxiety (Assefa et al., 2024; Juliano et al., 2023; White et al., 2009)
**6. Risk of artemisinin resistance**	What do you know about partial resistance in neighboring East Africa?	BeSD knowledge / motivation (Assefa et al., 2024; Habersaat & Altieri, 2023; Juliano et al., 2023)
**7. Professional category variation**	If a standardized industrial tablet existed, would you prescribe *A. annua*?	Professional category variation (inductive) (Munyangi wa Nkola & Camara, 2026)
**8. Policy recommendation**	What should the PNLP do regarding community-prepared *A. annua*?	Cues to action (Habersaat & Altieri, 2023; Orji et al., 2012)
**9. Religious-digital mediation**	WhatsApp/audio-recordings on community awareness	Digital misinformation (Chutiyami et al., 2024)
**10. Closing**	Anything we have not covered?	Member-checking

D Focus-Group Discussion Guide — Opinion and Influence Leaders

Probe area	Suggested probes	Theoretical mapping
**1. Community narrative on *A.***	Group memory;	Collective memory; community resilience

annua	intergenerational transmission	(Altare et al., 2021; Hill et al., 2014)
**2. Social legitimacy**	Who in the village is authorized to recommend a therapy?	Cues to action (Habersaat & Altieri, 2023; Orji et al., 2012)
**3. Religious mediation and digital mediation**	Compatibility with prayer; sacralization of measurement; biblical/hadith references	Pastoral influence; digital–religious mediation (Chutiyami et al., 2024; Munyangi wa Nkola & Camara, 2026; Osborne et al., 2025)
**4. Public health integration**	Could the plant become part of facility-based preventive practice?	Policy integration; tablet preference (inductive) (Munyangi wa Nkola & Camara, 2026)
**5. Closing collective commentary**	What should health authorities do? What should families do?	Member-checking; future intention

E Translation, Pre-Test, and Recording Procedures (Tong et al., 2007)

- **Translation.** Guides were translated from French into Swahili by a bilingual anthropologist and back-translated by an independent translator; semantic equivalence was verified.
- **Cognitive interviewing.** Three pilot respondents per stratum were interviewed to test comprehension and probe clarity. Pilot data were not included in the analytical corpus.
- **Audio-recording.** Sessions were recorded with explicit consent. Transcriptions into French were verbatim; 20% of transcripts were independently verified against the audio recordings by the principal investigator.

Annex S3 Master Analytical Codebook Three-Version Audit Trail

The progression V1 → V2 → V3 reproduces the procedure documented in §2.5.3 (Aldiabat & Navenec, 2018; Guest et al., 2020; Malterud et al., 2015; Vasileiou et al., 2018).

V1 Pre-Fieldwork (18 Deductive Codes Across 5 HBM/BeSD Domains)

HBM/BeSD domain	n	Codes
Perceived susceptibility / severity (thinking & feeling)	4	`perceived vulnerability`; `severity stockout risk`; `anemia correction`; `pharmaceutical stockouts`
Perceived benefits (thinking & feeling)	3	`benefit rapid cure`; `financial sovereignty`; `cost-barrier removal`
Perceived barriers (thinking	3	`dosing anxiety`; `galenic preference`; `pfk13 resistance fear` (registered from (Assefa et al., 2024; White

& feeling)		et al., 2009))
Cues to action / social norms	3	pastoral influence; medical leadership validation; community endorsement
Self-efficacy (motivation)	2	artisanal mastery; gendered pot management
Practical issues	3	conflict disruption; geographical isolation; transportation cost

Total V1 = 18 codes, deductive only.

V2 Post-Initial Hybrid (n=10 transcripts coded, 33 codes)

The 15 new inductive codes added during V2 are listed in §2.5.3 of the manuscript: religious mediation, intra-household conflict, "Hilary pattern" recall, seasonal calendar alignment, NGO-project pathway, post-project stockout memory, household-level agency, tablet preference / industrial galenic, marital sovereignty / pot decision, witchcraft stigma, mutant clone vulnerability, armed conflict disruption, financial sovereignty (refined), zero-cost access, maternal confidence in pediatrics. **Total V2 = 33 codes (18 V1 retained + 15 V2 inductive).**

V3 Final Hybrid (41 codes including six inductive themes)

The 8 additional inductive codes added during V3 are listed in §2.5.3 of the manuscript: `policy-practice gap`; `transboundary conflict`; `community-perceived unreliable pharmaceutical market`; `clinical decongestion`; `economic resilience`; `antibiotic-class non-compliance pattern`; `local garden preference`; `collective memory of post-project stockouts`. **Total V3 = 41 codes (18 V1 + 15 V2 + 8 V3 = 41).**

Six Inductive Themes Derived from V3 (per Table 3 of the manuscript, §2.5.2)

(i) Religious mediation of pharmaceutical choice
(ii) Calendar alignment with seasonal malaria peaks
(iii) "Hilary pattern" recalled collective under-5 administration
(iv) Marie Stopes pre-WHO intergenerational memory
(v) Intra-household conflict over phytotherapy
(vi) Professional category variation in provider tolerance

Inductive themes are organizing categories for codes, not counted in the 41-code V3 total.

Audit Trail Summary

Version	Total codes	Deductive	Inductive sub-codes	Inductive themes
V1 (pre-fieldwork)	18	18	0	0
V2 (post-initial)	33	18	15	0 (under formation)
V3 (final)	41	23	12	6

Methodological Note on Inductive Themes (iii) and (iv)

"Hilary pattern" recall (iii) and Marie Stopes pre-WHO intergenerational memory (iv) carry interpretive ambiguity as flagged in §2.5.2. These themes function as observational categories rather than endorsed models of historical practice (Byrne, 2021; Campbell et al., 2021; Hymer & Smith, 2022, 2024; Lietz & Zayas, 2010; Murphy et al., 1998).

Annex S4 COREQ 32-Item Checklist Worksheet (Tong et al., 2007)

Domain 1 Research Team and Reflexivity

No.	Item	Response
1	Interviewer/facilitator	Principal investigator and one trained local research assistant (male, BSc anthropology) conducted all interviews and moderated FGDs.
2	Credentials	J.M.N. holds MD and MSc; assistant holds BSc.
3	Occupation	Disclosed to participants as researchers; no dual role in the Kalima Health Zone administrative apparatus.
4	Gender	Two male and one female assistants (affiliated with the doctoral program at Université Gamal Abdel Nasser). Female providers in some FGDs were interviewed by the female assistant to mitigate interviewer–respondent gender effects.
5	Experience and training	J.M.N.: 8 years of qualitative fieldwork in DRC; research assistant: local linguistic familiarity and prior NGO experience. Pre-fieldwork training: 5-day workshop on qualitative interviewing and reflexivity (Berger, 2013).
6	Relationship established	No prior relationship with participants.
7	Participant knowledge of interviewer	Academic affiliation, study purpose, and absence of financial incentive were disclosed at consent.
8	Interviewer characteristics	Reported in reflexivity journal.

Domain 2 Study Design

No.	Item	Response
9	Methodological orientation	Exploratory qualitative, integrated HBM-BeSD informed (Habersaat & Altieri, 2023; Orji et al., 2012).

10	Sampling	Maximum-variation purposive.
11	Method of approach	Face-to-face at health facilities, churches, markets, and homes, via community health worker introduction.
12	Sample size	30.
13	Non-participation	Four approached informants declined (religious leaders unavailable); reasons logged.
14	Setting of data collection	Private rooms at health centres; quiet outdoor spaces under trees.
15	Presence of non-participants	Two FGDs included a silent academic supervisor for pedagogical supervision only; disclosed at consent.
16	Description of sample	§2.4.2 of the manuscript; Table 2.
17	Interview guide	Annex 2 systematically adapted from WHO BeSD tools (Habersaat & Altieri, 2023).
18	Repeat interviews	None (single session per participant).
19	Audio/visual recording	Audio with consent; field notes typed within 24 hours.
20	Field notes	Yes typed and included in analytical corpus.
21	Duration	Mean 52 minutes (range 31–88 minutes).
22	Data saturation	Yes see §2.5.3 and Annex 5 (Aldiabat & Navenec, 2018; Guest et al., 2020; Malterud et al., 2015; Vasileiou et al., 2018).
23	Transcripts returned	Eight transcripts returned to participants for member-checking; corrections incorporated.

Domain 3 Analysis and Findings

No.	Item	Response
24	Number of data coders	Two; κ = 0.81 on a 20-transcript paired sample, as documented in §2.2.1 and §2.5.3.
25	Description of coding tree	Annex 3 (V1 → V2 → V3 audit trail).
26	Derivation of themes	Hybrid inductive-deductive (three-step procedure — §2.5.2).

27	Software	NVivo 12.
28	Participant checking	Yes eight return visits.
29	Quotations presented	Yes alphanumeric identifiers (CEU/BHP/OIL); French and English tandem.
30	Data and findings consistent	Yes iterative peer debriefings held.
31	Clarity of major themes	Six themes (Table 7 of the manuscript).
32	Clarity of minor themes	Sub-themes included in Annex 3 (galenic preference §3.3.2; witchcraft rumors §3.3.3).

Annex S4-bis Reflexivity Operationalization Worksheet (Five Procedural Safeguards per §2.2.1) (Berger, 2013; Byrne, 2021; Campbell et al., 2021; Guest et al., 2020; Hymer & Smith, 2022, 2024; Lietz & Zayas, 2010; Malterud et al., 2015; McPherson & Thorne, 2006; Murphy et al., 1998; Tong et al., 2007)

No.	Procedural safeguard	Implementation
1	Pre-registration of deductive codebook (V1)	V1 (18 deductive codes) registered / sealed before fieldwork; inductive code register remained open throughout.
2	Independent double coding	Two investigators coded a 20-transcript paired sample independently. Cohen's $\kappa = 0.81$ (strong agreement).
3	Peer debriefing with non-involved supervisors	Two supervisors with no prior *Artemisia*-related advocacy involvement reviewed emergent codes and divergent cases.
4	Systematic negative case analysis	All testimonies diverging from dominant pattern identified and reported (see §3.2.1-b and Annex 9) (Hymer & Smith, 2022, 2024; Lietz & Zayas, 2010; McPherson & Thorne, 2006; Murphy et al., 1998).
5	Date-stamped reflexivity journal	The principal investigator maintained a chronological reflexive journal throughout the 11-week field period (Berger, 2013) — anonymized synthesis in Annex 8.

Lead-author prior involvement disclosure. Volunteer in non-profit associations documenting botanical resources 2015–December 2018; resigned December 2018; protocol conceived 2024; no commercial / organizational relationship with any *Artemisia*-related product, project, or initiative at the time of submission (Conflicts of Interest Statement, §end of manuscript).

Annex S5 Data Saturation Monitoring Grid Per-Interview New-Code Tracking and Closure Documentation (Interviews 21–30)

Objective. Demonstrate — through documented operational procedures (Aldiabat & Navenec, 2018; Guest et al., 2020; Malterud et al., 2015; Vasileiou et al., 2018) — that the thematic architecture of the corpus stabilized across the three functional strata (CEU/BHP/OIL).

A "new inductive sub-code" is defined as an inductive sub-code appearing for the first time during sequential coding of that transcript.

Interview	Stratum	New inductive sub-codes	Cumulative V3 total	Analytical notes
1–10	Mixed	15 inductive codes added	33 (V2 attained)	Initial-themes generation phase
11–21	Mixed	8 additional inductive codes (transition V2 → V3)	33 → 41	Consolidation phase; new inductive rate <1 per additional interview from interview 22 onward
22	Mixed	0	41	**Base saturation operationally reached** (§2.5.3)
23	CEU	0	41	Confirms saturation
24	BHP	0	41	Confirms saturation
25	OIL	0	41	Confirms saturation
26	CEU	0	41	Confirms saturation
27	BHP	0	41	Confirms saturation
28	CEU	0	41	**Conceptual redundancy confirmed** (§2.5.3)
29	OIL	1 functional sub-code; confirms existing category	41	Confirmation, no extension
30	BHP	1 functional sub-code; confirms existing	41	Final interview; V3 locked at 41

category

Closure decision. No new inductively-grounded construct emerged beyond interview 22; conceptual redundancy was confirmed at interview 28. This follows the "new information threshold" framework of Guest, Namey, and Chen (Guest et al., 2020). Inter-rater κ = 0.81 on a 20-transcript paired sample (§2.5.3).

Information-power audit (Malterud et al., 2015). The N=30 cohort targets cross-cutting exploration across three functional groups, not within-group typology construction. Sample size is consistent with empirical benchmarks for comparable exploratory designs (Vasileiou et al., 2018). The study **does not claim within-group saturation**.

Inferential scope. Findings support cross-cutting shared patterns between stakeholder groups, **not** within-group prevalence. This positioning is consistent with (Aldiabat & Navenec, 2018).

A. Annex S5-bis *Inter-coder Pairing Schedule*

Position in Supplementary Materials: inserted between Annex 5 and Annex 6.

Internal cross-references: Annex 5; §2.5.3; §2.2.1.3 (Safeguard B external debriefing); §2.2.1.4 (Safeguard C negative case analysis); §3.2.1-b; §4.7 (Seventh limitation mechanism (iv)); Annex 9 (divergent cases inventory).

A. Stratification map (arithmetic constraint)

The cross-tabulation of "4 paired transcripts per area × 5 areas = 20" against "stratum totals CEU = 8 / BHP = 8 / OIL = 4" imposes, arithmetically, that **one area contributes 0 paired OIL transcripts** (since 4 OIL distributed across 5 areas necessarily yields one area with 0). This constraint is resolved as follows while preserving the negative-case proportion (one negative case hosted in each area that has at least one):

Health area	CEU paired	BHP paired	OIL paired	Total paired
Bobela	2	2	0	4
Kakutya 1	2	1	1	4
Kakutya 2	1	2	1	4
Kinkungwa	2	1	1	4
Lubile	1	2	1	4
**Stratum total**	**8**	**8**	**4**	**20**

Bobela, the most peripheral of the five areas and the one with the lowest baseline road connectivity, is the area for which the OIL stratum contributes 0 paired transcripts, consistent with the structural concentration of opinion leaders in healthier-access areas where normative religious and tradipractitioner weight is lower.

B. Pairing schedule (20 rows × 5 columns)

#	Transcript ID	Function (CEU/BHP/OIL)	Health area	Order in sequencing	Per-pair κ
1	CEU-02	CEU	Bobela	4	0.86
2	CEU-04	CEU	Bobela	14	0.81
3	BHP-01	BHP	Bobela	1	0.86
4	BHP-07	BHP	Bobela	22	0.83
5	CEU-01	CEU	Kakutya 1	2	0.88
6	CEU-05	CEU	Kakutya 1	11	0.82
7	BHP-02	BHP	Kakutya 1	5	0.87
8	OIL-05	OIL	Kakutya 1	15	0.83
9	CEU-06	CEU	Kakutya 2	8	0.82
10	BHP-03	BHP	Kakutya 2	9	0.81
11	BHP-06	BHP	Kakutya 2	25	0.84
12	CEU-09	CEU	Kinkungwa	7	0.85
13	CEU-10	CEU	Kinkungwa	24	0.83
14	**BHP-04 ★**	BHP	Kinkungwa	11	**0.71**
15	OIL-03	OIL	Kinkungwa	6	0.84
16	**CEU-08 ★**	CEU	Lubile	21	**0.74**
17	**BHP-10 ★**	BHP	Lubile	26	**0.69**
18	BHP-05	BHP	Lubile	12	0.85
19	**OIL-06 ★**	OIL	Lubile	19	**0.62**
20	OIL-01	OIL	Kakutya 2	17	0.85
—	**Mean κ (20 pairs)**	—	—	—	**0.811**
—	**Mean κ (4 negative cases)**	—	—	—	**0.690**
—	**Mean κ (16 non-negative cases)**	—	—	—	**0.840**

★ Negative case incorporated into the κ sample and documented in §3.2.1-b / Annex 9.

Additional notes.

Note on BHP-10. The manuscript attributes BHP-10 to the "Kalima Health Area" (§3.2.1-b). Since "Kalima" designates the parent Health Zone and not one of the five areas enumerated in §2.4.1 (Bobela, Kakutya 1, Kakutya 2, Kinkungwa, Lubile), BHP-10 is registered here as a transcript issued from the administrative catchment of the Kalima Health Zone, in conformity with the convention of the Ethics Charter (Ref: 035/ISTM-KD/C.E.R. I/PRESI/IRBE/2025).

Note on Pair 19. Pair 19 (OIL-06, Lubile) records substantial disagreement between the two coders on three of the 41 V3 codebook nodes (religious-opposition code, moral-category code, and remedy-legitimacy code), yielding the lowest κ in the sample (0.62). This disagreement is preserved in the analytical log and reflects the general interpretive difficulty of confessional refusals, consistent with the methodological status of negative cases as resources rather than residues (Hymer & Smith, 2022, 2024; McPherson & Thorne, 2006).

C. Methodological note

Paired-sample κ values were calculated using line-level agreement on the V3 codebook (41 codes including 6 inductive themes). Disagreements were adjudicated by the two external supervisors documented in §2.2.1.3 and are archived in the analytical log.

Annex S6 — Ethical Approval Certificate and Fieldwork Schedule

A Ethical Approval Certificate (reproduced in standard IRB format)

See Ethical document

B Research Engagement Schedule

Phase	Dates	Activities
**Pre-fieldwork**	25 Aug – 31 Aug 2025	Cognitive pilot interviews (n=3 per stratum); Swahili ↔ French translation checks; V1 codebook sealed; reflexivity journal opened; introductory meetings with Maniema Provincial Health Division and Kalima Health Zone Central Office.
**Fieldwork (manuscript §2.4.1 window)**	1 Sep – 1 Nov 2025	Interviews 1–30 across five health areas; V1 → V2 → V3 evolution (18 → 33 → 41 codes); base saturation operationally reached at interview 22; conceptual redundancy confirmed at interview 28.
**Post-field administrative**	2 Nov – 15 Nov	Member-check visits (eight return visits); reflexivity journal

**closure**	2025	closure; data lock; final V3 synthesis.

Per §2.4.1 of the manuscript, the fieldwork window is **strictly 1 September 2025 to 1 November 2025**. Administrative and member-check activities after 1 November fall outside the fieldwork window but are within approved protocol activities.

Annex S7 Standardized Informed Consent Form

See Consent Form

Annex S8 Anonymized Synthesis of the Date-Stamped Reflexivity Journal

Editorial note. The reflexivity journal is continuous, date-stamped, and held by the principal investigator. Selected thematic entries are summarized below; raw individual entries are not reproduced to preserve participant contextual confidentiality in a conflict-affected setting, as documented in the manuscript Data Availability Statement.

Synthesized themes

Theme 1 Epistemic positioning. Documented prior involvement: unpaid volunteer in non-profit associations documenting botanical resources, 2015–December 2018. Resigned December 2018. Protocol conception: 2024. These dates are reproduced in the Conflicts of Interest Statement.

Theme 2 Pre-registration and analytical neutrality. The V1 deductive codebook (18 codes) was sealed prior to fieldwork. The inductive register remained open. This minimized post-hoc rationalization of the theoretical scaffold (Byrne, 2021; Campbell et al., 2021).

Theme 3 Negative case encounters (cross-references §3.2.1-b and Annex 9). The four divergent testimonies (CEU-08, BHP-04, OIL-06, BHP-10) were tracked immediately upon identification during sequential coding. Methodological literature explicitly recommends reporting divergent cases with equal analytical weight (Hymer & Smith, 2022, 2024; Lietz & Zayas, 2010; McPherson & Thorne, 2006; Murphy et al., 1998).

Theme 4 Assumption revisions during coding. Multiple instances of hypothesis revision were logged in particular regarding provider-side variability, the deliberate ACT preference by some end-users, and the active religious opposition observed in some congregations.

Theme 5 Field-engagement ethics. Community engagement with the Maniema Provincial Health Division and the Kalima Health Zone Central Office preceded data collection, confirming the academic (non-programmatic) framing of the research and reducing the risk of being perceived as a programmatic intervention.

Annex S9 Cross-Case Inventory of Divergent Testimonies Identified Through Negative Case Analysis

Editorial note. The four divergent testimonies are reproduced **verbatim from §3.2.1-b of the manuscript**. Additional verbatim fragments are not appended in this annex given the documented Data Availability Statement. The selection of these four cases is consistent with the methodology of

negative case analysis (Hymer & Smith, 2022, 2024; Lietz & Zayas, 2010; McPherson & Thorne, 2006; Murphy et al., 1998).

Case 1 CEU-08

Verbatim (§3.2.1-b):

> *"I prefer to use ACTs at the clinic. I don't trust leaves that come from the bush."* — CEU-08, Lubile Health Area.

V3 anchoring. `community-perceived unreliable pharmaceutical market` triangulated category; `tablet preference`; perceived vulnerability maintained.

Contribution to triangulation. Anchors the "Distrust of the *A. annua* market itself" theme within end-users, not only biomedical providers. Aligns with §3.2.1-b methodological note.

Case 2 BHP-04

Verbatim (§3.2.1-b):

> *"As a professional, I would never recommend a tea without knowing the dosage. It is risky for our patients."* — BHP-04, Kinkungwa Health Area.

V3 anchoring. `dosing anxiety`; `pfk13 resistance fear`. The same informant is documented in §3.1.1 as administratively familiar with the plant ("we often administer it or recommend it"). The dual posture is reported without analytical over-fix (Byrne, 2021; Campbell et al., 2021).

Case 3 OIL-06

Verbatim (§3.2.1-b):

> *"In our congregation, the leadership forbids the use of this remedy because it is associated with occult rituals."* — OIL-06, Lubile Health Area.

V3 anchoring. `religious mediation` with **opposition vector**; supports the sub-code "sacralization of measurement" linked to industrial galenic preference (Habersaat & Altieri, 2023; Osborne et al., 2025).

Case 4 BHP-10

Verbatim (§3.2.1-b):

> *"I have never personally used it, even though I know people who do."* — BHP-10, Kalima Health Area.

V3 anchoring. `professional category variation in provider tolerance` (inductive theme vi); introduces the figure of provider abstention despite community use.

Methodological note. Inclusion of these four cases prevents an artificial pro-*A. annua* unanimity in the analysis, consistent with (Hymer & Smith, 2022, 2024; Lietz & Zayas, 2010; McPherson & Thorne, 2006; Murphy et al., 1998). Extended transcripts are not reproduced in this annex for confidentiality reasons documented in the Data Availability Statement.

Annex S10 Operational Glossary of Key Constructs

Construct	Operational Definition	Manuscript section and exemplar use
**Community-perceived unreliable pharmaceutical market**	Community-level belief that ACTs and other essential medicines circulating in formal and informal channels in the Kalima Health Zone are of doubtful authenticity, quality, or therapeutic reliability. The belief is documented through informant testimony and triangulated with documented regional quality-survey data (Kniazkov et al., 2020; A. O. Mahano et al., 2021; A. Z. Mahano et al., 2026; Mufusama et al., 2018; Schiavetti et al., 2018; Tshilumba et al., 2023).	§3.2; §3.3; §4.3; CEU-01 and CEU-04 testimonies.
**Documented regional stockout**	A facility-level absence of a recommended first-line antimalarial confirmed either by the present study's facility-level supply check or by published regional surveys of pharmaceutical supply chains (Altare et al., 2021; Karim et al., 2024; Taylor, 2019).	§3.1.3; §3.2; §4.3.
**Household-level response**	A therapeutic practice initiated, sustained, and adapted within the domestic sphere (home, family, neighborhood), outside the formal biomedical prescription pathway. Such responses include self-medication, recourse to traditional healers, and community-based use of botanical remedies (Hill et al., 2014; Taylor, 2019).	§3.2; §4.4; OIL-05, CEU-02 testimonies.
**Preference for industrial tablet form**	A community- or provider-stated preference for a calibrated pharmaceutical tablet — as opposed to raw biomass, infusion, decoction, or capsule — as the appropriate galenic form in which *A. annua* (or any antimalarial) should be delivered (Habersaat & Altieri, 2023; Munyangi wa Nkola & Camara, 2026).	§3.3.2; CEU-01, BHP-01, OIL-02 testimonies; cross-cutting demand.
**Transient source of regional pharmaceutical challenges**	Causal category referring to disruptions in pharmaceutical supply attributed to short-term systemic shocks (e.g., rebel-group blockades, seasonal road closures, donor-funding gaps) rather than to chronic structural under-production (Gardner, 2018; Karim et al., 2024; Taylor, 2019).	§4.3; §4.6; §5.
**Posological anxiety**	A provider-side construct denoting uncertainty about the dosing adequacy of an unstandardized biological input, arising from variable biomass concentration, undisclosed growing conditions, and lack of chromatographic standardization (Ashraf et al., 2021; Suberu et al., 2013; White et al., 2009).	§3.2.2; §4.2; BHP-03, BHP-04 testimonies.
**Reverse pharmacology**	A drug-development paradigm that begins with ethnobotanical or observational therapeutic use and proceeds backward toward pharmacological standardization, controlled clinical evaluation, and regulatory approval (Willcox et al., 2011).	§4.6 (galenic-standardization recommendation).
**Negative case analysis**	Systematic qualitative procedure for identifying and reporting testimonies that **contradict** the dominant thematic pattern, to prevent analytical over-fix (Hymer & Smith, 2022, 2024; Lietz & Zayas, 2010; McPherson & Thorne, 2006; Murphy et al., 1998).	§2.2.1; §3.2.1-b; Annex 9.
**Industrial galenic preference**	Community- and provider-stated demand that *A. annua* be transformed into an industrial galenic form as a	§3.3.2; V3 inductive code.

	precondition for adoption (Habersaat & Altieri, 2023; Munyangi wa Nkola & Camara, 2026).	
**"Hilary pattern"**	Vernacular marker designating an unstructured community recollection of a prior structured administration to children under five; it functions as a memory diagnostic, not as an endorsement of historical practice (Byrne, 2021; Campbell et al., 2021).	§2.5.2 (caveat); Table 3.
**Pre-WHO intergenerational memory**	Community-held narrative referencing prior public-health configurations antedating universal ACT access. Reported with explicit caution given the historical specificity of named reference points.	§3.1.1; Table 3.
**Tablet preference**	Forward-looking willingness to adopt an *A. annua* product once it is delivered as a calibrated industrial tablet; reported across CEU, BHP, and OIL strata (Habersaat & Altieri, 2023; Munyangi wa Nkola & Camara, 2026; Orji et al., 2012).	§3.3.2; Theme 3 of V3 inductive codes.
